# Public health risk assessment of rodent-driven hantavirus spillover in Europe using a stochastic delay model

**DOI:** 10.3389/fpubh.2026.1880617

**Published:** 2026-07-28

**Authors:** Ali Raza, Umar Shafique, Marek Lampart, Dumitru Baleanu, Emad Fadhal, Hadil Alhazmi

**Affiliations:** 1 IT4Innovations, VSB–Technical University of Ostrava, Ostrava, Czechia; 2 Faculty of Electrical Engineering and Computer Science, VSB–Technical University of Ostrava, Ostrava, Czechia; 3 Department of Computer Science and Mathematics, Lebanese American University, Beirut, Lebanon; 4 Department of Mathematics and Statistics, College of Science, King Faisal University, Al Ahsa, Saudi Arabia; 5 Department of Mathematical Sciences, College of Science, Princess Nourah bint Abdulrahman University, Riyadh, Saudi Arabia

**Keywords:** Brownian motion, extinction and persistence, hantavirus, human spillover infection, numerical simulation, public health risk, rodent-borne transmission, sir–sir model

## Abstract

**Introduction:**

Hantavirus is a rodent-borne zoonotic disease in which rodents serve as the primary reservoir, while humans acquire infection through spillover exposure. To better understand the effects of environmental variability and delayed disease dynamics on transmission, this study develops a stochastic delay differential model that incorporates biologically relevant features of hantavirus spread.

**Methods:**

An SIR–SIR transmission framework is formulated for interacting rodent and human populations. The deterministic model is extended by incorporating Brownian stochastic perturbations, discrete time delays, and exponential delay-survival effects to capture incubation, survival, and environmental uncertainty. The qualitative properties of the model, including positivity, boundedness, feasibility, and well-posedness, are established. Disease-free and endemic equilibria are derived, and both deterministic and stochastic reproduction numbers are obtained. Extinction and persistence conditions are investigated using stochastic threshold analysis. Numerical simulations are carried out using the Euler–Maruyama, stochastic Runge–Kutta, and stochastic nonstandard finite difference (SNSFD) methods.

**Results:**

The analysis shows that hantavirus persistence is primarily governed by rodent-to-rodent transmission, whereas human infections arise through spillover from infected rodents. Stochastic perturbations and delay-survival effects reduce the effective reproduction threshold under certain conditions and can promote disease extinction. Theoretical findings are supported by numerical simulations, which demonstrate that all three numerical methods produce consistent solutions for small time steps. However, the SNSFD scheme preserves positivity and exhibits superior numerical stability under larger time steps and stronger stochastic perturbations. Reported hantavirus updates from 2026 are incorporated to motivate the model and emphasize the continued importance of reservoir-driven transmission dynamics.

**Discussion:**

The proposed stochastic delay model provides a mathematically rigorous and biologically realistic framework for studying hantavirus transmission. The results highlight that controlling infection within the rodent reservoir is the most effective strategy for reducing disease persistence and limiting human spillover. The improved stability of the SNSFD method also makes it a suitable computational approach for simulating stochastic epidemic systems with delays.

## 1 Introduction

According to the latest WHO update on 12 May 2026, a multi-country hantavirus cluster linked to the MV *Hondius* cruise ship reported 11 cases, including three deaths. Among these cases, nine were confirmed as Andes virus infections and two were classified as probable cases. WHO noted that all reported cases occurred among passengers or crew members, and no additional deaths had been reported since WHO was first informed of the cluster on 2 May 2026 (1). Hantavirus remains an important emerging zoonotic pathogen, reflecting the breadth of its reservoir, severity of clinical manifestations and ongoing public health importance, as evidenced by recent literature. D'Souza and Patel surveyed hantaviruses of the New World and their relevance to biodefense and viewed them as serious diseases potentially from a natural source in rodents, but are difficult to cultivate and disseminate in practice (2). Likewise, in Canada, Warner et al. investigated hantavirus cardiopulmonary syndrome and found that the deer mouse was the primary reservoir host, and Sin Nombre virus was the leading cause of human disease (3). The authors also mentioned that rodent exposure, environmental modification, and poor surveillance are significant risk factors for zoonotic spillover of hantavirus and discussed the issue in the wider context of zoonotic spillover in general (4). Recent research has enhanced understanding of the molecular and virological mechanisms of hantavirus replication, structure, and immune evasion. Meier et al. gave an updated structural virology review of the hantavirus replication cycle, elucidating the mechanisms of viral entry, genome replication, assembly, and release, as well as how these mechanisms relate to antiviral and vaccine development targets (5). Zhang et al. explained the ability of hantaviruses to disrupt innate antiviral signaling, which contributes to the ability to persistently infect reservoir hosts that are typically healthy and to cause severe disease in humans (6). Engdahl et al. also made a contribution to this field, by mapping human antibody responses against hantavirus antigens, which can aid in vaccine development and serological diagnostics (7). Studies conducted during this period include transmission and outbreak studies and underscore the ongoing need for surveillance and early detection. Toledo et al. conducted a systematic review of the evidence and found that Andes virus might be transmitted from person-to-person, but more controlled studies are needed (8). Sah et al. reviewed recent hantavirus outbreaks and pointed out that there are still no specific approved treatments, high fatality risks, and delayed diagnosis of hantavirus, which are still major clinical challenges (9). Goodfellow et al. described an infection with an orthohantavirus in humans in Michigan, USA, indicating that physicians should be aware of the possibility of a hantavirus infection beyond the classic “hotspot” areas where exposure to hantaviruses is known to occur and when signs and symptoms are consistent with exposure history. During this time, too, there has been a growth of clinical literature (10). Afzal et al. examined the available therapeutic options such as antiviral agents, immune-based treatments, and vaccine candidates, but found that supportive care is the primary management approach due to the lack of a widely accepted targeted therapy for hantavirus (11). Vial et al. summarized clinical features, diagnosis, management, and prevention of hantavirus infection in humans, describing the disease as a zoonotic viral haemorrhagic infection with significant clinical relevance, an emerging disease (12). Wu et al. emphasized the importance of hantavirus in children with acute kidney injury and thrombocytopenia, suggesting that hantavirus should be included in the differential diagnosis of pediatric acute kidney injury patients with thrombocytopenia (13). Hantavirus distribution in the region/ecology is influenced by reservoir ecology, geography, land use and human exposure. Chen et al. reviewed zoonotic members of the family *Hantaviridae* of global health importance and highlighted the importance of integrated ecological, molecular and epidemiological surveillance (14). Lupusoru et al. summarized the situation with hantavirus infections in South-Eastern Europe, particularly in relation to Puumala and Dobrava viruses, and associated the distribution of reservoirs with the patterns of disease in the region (15). Mello et al. studied the expansion of hantavirus within natural host populations and linked the ecological changes, host distribution, and spillover risk (16). These population level and seroepidemiological studies also show that exposure to hantavirus is not uniform among regions or occupational groups. Whitmer et al. investigated the prevalence and genotype distribution of human orthohantavirus disease in the United States, identifying regional clustering and gaps that affect case detection (17). Tortosa et al. performed a systematic review and meta-analysis of hantavirus seroprevalence in non-epidemic settings and revealed that exposures vary by geography, occupation and study population (18). Gubareva et al. studied the prevalence of hantavirus antibodies in high-risk adults in Western Kazakhstan and determined occupational and environmental exposures that potentially could be factors in hantavirus infection (19). Studies of hantavirus reservoirs and wildlife have recently documented the continued importance of animal surveillance for preventing hantavirus. Goodfellow et al. studied the circulation and shedding of human-pathogenic hantaviruses in various rodent reservoirs, which helped their study to clarify the environmental exposure risk for hantaviruses in infected hosts (20). Guo et al. performed a systematic review and meta-analysis of the prevalence of hantaviruses in small mammals of Southeast Asia, and recommended for expanding the surveillance of wildlife for the detection of zoonotic risk in advance (21).

Stochastic delay modeling provides a framework for describing real-world systems affected by both random environmental variations and delayed responses. By including uncertainty and time-lag terms in the mathematical model, it reflects system behavior more accurately. This approach helps improve prediction and gives deeper insight into complex dynamic processes.

The paper is organized as follows. Section 1 presents the introduction and background of the study. Section 2 describes the preliminary concepts from stochastic analysis and stochastic delay differential equations. Section 3 formulates the stochastic delay hantavirus model using susceptible, infected, and recovered human populations together with susceptible, infected, and recovered rodent populations. Section 4 discusses the qualitative analysis, including positivity, boundedness, disease-free and endemic equilibria, reproduction numbers, extinction, and persistence. Section 5 presents the real reported hantavirus data and its connection with the model. Section 6 introduces the numerical schemes, including Euler–Maruyama, stochastic Runge–Kutta, and stochastic NSFD methods. Section 7 provides graphical simulations and interpretation of the numerical results. Finally, Section 8 gives the concluding remarks and possible future extensions.

## 2 Preliminaries

In this section, we recall some basic concepts from stochastic analysis and stochastic delay differential equations that are used in the formulation and qualitative analysis of the stochastic delay hantavirus model.

**Definition 1** (Brownian motion (22, 23)). A real-valued stochastic process {*W*(*t*), *t* ≥ 0} defined on a complete probability space (Ω, ℱ, ℙ) is called a standard Brownian motion if it satisfies the following properties:

1. *W*(0) = 0 almost surely;
2. *W*(*t*) − *W*(*s*) is independent of ℱ_*s*_ for all 0 ≤ *s* < *t*;
3. *W*(*t*) − *W*(*s*) ~ 𝒩(0, *t* − *s*) for all 0 ≤ *s* < *t*;
4. *W*(*t*) has continuous sample paths almost surely.

**Definition 2** (Stochastic delay differential equation (23)). A stochastic delay differential equation is an equation in which the present rate of change of a stochastic process depends on both its current state and its past state. A general SDDE can be written as


dX(t)=f(t,X(t),X(t-τ))dt+g(t,X(t),X(t-τ))dW(t),

where τ > 0 is a fixed delay, *f* is the drift coefficient, *g* is the diffusion coefficient, and *W*(*t*) is a standard Brownian motion.

**Definition 3** (Initial history function (23)). For an SDDE with delay τ > 0, the solution is determined not only by the value at *t* = 0 but also by its past values on the interval [−τ, 0]. Thus, the initial condition is prescribed as a continuous history function


X(θ)=ϕ(θ),  θ∈[-τ,0],

where


$$\phi \in C\left(\left[-\tau ,0\right],{\mathbb{R}}_{+}^{n}\right).$$

Here, $C\left(\left[-\tau ,0\right],{\mathbb{R}}_{+}^{n}\right)$ denotes the space of continuous functions from [−τ, 0] into ${\mathbb{R}}_{+}^{n}$.

**Definition 4** (Positive solution (22, 23)). A solution *X*(*t*) of an SDDE is said to be positive if


X(t)>0 almost surely for all t≥0,

whenever the initial history satisfies


X(θ)=ϕ(θ)>0,  θ∈[-τ,0].

In biological models, positivity ensures that population variables remain meaningful.

**Definition 5** (Mean-square stability (23)). The disease-free state is said to be mean-square stable with respect to an infected compartment *I*(*t*) if


$$\underset{t\to \infty }{\lim}𝔼\left[I^{2}\left(t\right)\right]=0.$$

If, in addition, there exist constants *K* > 0 and λ > 0 such that


$$𝔼\left[I^{2}\left(t\right)\right]\leq Ke^{-\lambda t},$$

then the disease-free state is said to be exponentially stable in the mean-square sense.

**Definition 6** (Non-negative solution (22, 23)). A solution *X*(*t*) of an SDDE is said to be non-negative if


$$X\left(t\right)\in {\mathbb{R}}_{+}^{n}\text{ almost surely for all }t\geq 0,$$

whenever the initial history satisfies


$$X\left(\theta \right)=\phi \left(\theta \right)\in {\mathbb{R}}_{+}^{n},\;\theta \in \left[-\tau ,0\right].$$

In biological models, non-negativity ensures that population variables remain meaningful.

## 3 Model formulation

Following the deterministic hantavirus transmission structure between humans and rodents, we divide the human population into susceptible, infected, and recovered classes, denoted by *S*_*H*_(*t*), *I*_*H*_(*t*), and *R*_*H*_(*t*), respectively. Similarly, the rodent population is divided into susceptible, infected, and recovered classes, denoted by *S*_*R*_(*t*), *I*_*R*_(*t*), and *R*_*R*_(*t*), respectively. Thus,


$$N_{H}\left(t\right)=S_{H}\left(t\right)+I_{H}\left(t\right)+R_{H}\left(t\right),\;N_{R}\left(t\right)=S_{R}\left(t\right)+I_{R}\left(t\right)+R_{R}\left(t\right).$$

The deterministic model assumes that hantavirus is transmitted from infected rodents to susceptible humans and among rodents, while human-to-human and human-to-rodent transmission are neglected. To make the model more realistic, we extend the system by including random environmental fluctuations, incubation or response delays, and exponential survival effects during the delay period.

Let τ_*H*_ > 0 denote the delay associated with transmission from infected rodents to humans, and let τ_*R*_ > 0 denote the delay associated with transmission among rodents. The terms $e^{-\eta_{H}\tau_{H}}$ and $e^{-\eta_{R}\tau_{R}}$ represent exponential survival or persistence factors during the delay periods, where η_*H*_ and η_*R*_ are non-negative decay parameters. The stochastic perturbations are introduced through independent Brownian motions ${\left\{W_{i}\left(t\right)\right\}}_{i=1}^{6}$, and σ_*i*_ represent the corresponding noise intensities. Table 1 presents the state variables and parameters used in the stochastic delay hantavirus model.

**Table 1:** State variables and parameters of the stochastic delay hantavirus model.

Symbol	Description
*S*_*H*_(*t*)	Susceptible human population
*I*_*H*_(*t*)	Hantavirus-infected human population
*R*_*H*_(*t*)	Recovered human population
*S*_*R*_(*t*)	Susceptible rodent population
*I*_*R*_(*t*)	Infected rodent population
*R*_*R*_(*t*)	Recovered rodent population
*N*_*H*_(*t*)	Total human population, *S*_*H*_ + *I*_*H*_ + *R*_*H*_
*N*_*R*_(*t*)	Total rodent population, *S*_*R*_ + *I*_*R*_ + *R*_*R*_
Γ_*H*_	Recruitment rate of humans
Γ_*R*_	Recruitment rate of rodents
*b*	Effective contact rate between humans and rodents
β_*H*_	Transmission probability from infected rodents to humans
β_*R*_	Transmission rate among rodents
γ_*H*_	Recovery rate of infected humans
γ_*R*_	Recovery rate of infected rodents
μ_*H*_	Natural death rate of humans
μ_*R*_	Natural death rate of rodents
τ_*H*_	Delay in rodent-to-human infection
τ_*R*_	Delay in rodent-to-rodent infection
η_*H*_	Exponential decay rate during rodent-to-human delay
η_*R*_	Exponential decay rate during rodent-to-rodent delay
σ_*i*_	Intensity of stochastic perturbation, *i* = 1, …, 6
*W*_*i*_(*t*)	Independent standard Brownian motions, *i* = 1, …, 6

The stochastic delay hantavirus model with exponential delay effects is formulated as follows:


$$\begin{array}{l} \begin{array}{r} dS_{H}\left(t\right)=[\Gamma_{H}-b\beta_{H}S_{H}\left(t-\tau_{H}\right)I_{R}\left(t-\tau_{H}\right)e^{-\eta_{H}\tau_{H}}\\ -\mu_{H}S_{H}\left(t\right)]dt+\sigma_{1}S_{H}\left(t\right)dW_{1}\left(t\right), \end{array} \end{array}$$ (1)

$$\begin{array}{l} \begin{array}{r} dI_{H}\left(t\right)=[b\beta_{H}S_{H}\left(t-\tau_{H}\right)I_{R}\left(t-\tau_{H}\right)e^{-\eta_{H}\tau_{H}}\\ -\left(\mu_{H}+\gamma_{H}\right)I_{H}\left(t\right)]dt+\sigma_{2}I_{H}\left(t\right)dW_{2}\left(t\right), \end{array} \end{array}$$ (2)

$$\begin{array}{l} \begin{array}{r} dR_{H}\left(t\right)=[\gamma_{H}I_{H}\left(t\right)-\mu_{H}R_{H}\left(t\right)]dt+\sigma_{3}R_{H}\left(t\right)dW_{3}\left(t\right), \end{array} \end{array}$$ (3)

$$\begin{array}{r} dS_{R}\left(t\right)=[\Gamma_{R}-\beta_{R}S_{R}\left(t-\tau_{R}\right)I_{R}\left(t-\tau_{R}\right)e^{-\eta_{R}\tau_{R}}-\mu_{R}S_{R}\left(t\right)]\\ dt+\sigma_{4}S_{R}\left(t\right)dW_{4}\left(t\right), \end{array}$$ (4)

$$\begin{array}{r} dI_{R}\left(t\right)=[\beta_{R}S_{R}\left(t-\tau_{R}\right)I_{R}\left(t-\tau_{R}\right)e^{-\eta_{R}\tau_{R}}-\left(\mu_{R}+\gamma_{R}\right)I_{R}\left(t\right)]\\ dt+\sigma_{5}I_{R}\left(t\right)dW_{5}\left(t\right), \end{array}$$ (5)

$$\begin{array}{l} dR_{R}\left(t\right)=[\gamma_{R}I_{R}\left(t\right)-\mu_{R}R_{R}\left(t\right)]dt+\sigma_{6}R_{R}\left(t\right)dW_{6}\left(t\right). \end{array}$$ (6)

In this system, the infection term $b\beta_{H}S_{H}\left(t-\tau_{H}\right)I_{R}\left(t-\tau_{H}\right)e^{-\eta_{H}\tau_{H}}$ describes delayed hantavirus transmission from infected rodents to humans. The exponential factor $e^{-\eta_{H}\tau_{H}}$ accounts for the reduction in effective transmission caused by mortality, environmental decay, or loss of infectivity during the delay period. Similarly, $\beta_{R}S_{R}\left(t-\tau_{R}\right)I_{R}\left(t-\tau_{R}\right)e^{-\eta_{R}\tau_{R}}$ represents delayed rodent-to-rodent transmission after accounting for exponential survival or persistence during the delay period.

Since system (Equations 1–6) is a stochastic delay differential equation, its solution depends on the previous population history over the interval [−τ, 0], where


$$\tau =\max\left\{\tau_{H},\tau_{R}\right\}.$$

Therefore, the initial conditions are prescribed as


$$S_{H}\left(\theta \right)=\phi_{1}\left(\theta \right),\;I_{H}\left(\theta \right)=\phi_{2}\left(\theta \right),\;R_{H}\left(\theta \right)=\phi_{3}\left(\theta \right),$$

$$S_{R}\left(\theta \right)=\phi_{4}\left(\theta \right),\;I_{R}\left(\theta \right)=\phi_{5}\left(\theta \right),\;R_{R}\left(\theta \right)=\phi_{6}\left(\theta \right),\;\theta \in \left[-\tau ,0\right].$$

Here, each scalar history function satisfies


$$\phi_{i}\in C\left(\left[-\tau ,0\right],{\mathbb{R}}_{+}\right),\;i=1,2,\ldots ,6,$$

and the corresponding vector-valued initial history function is given by


$$\phi =\left(\phi_{1},\phi_{2},\phi_{3},\phi_{4},\phi_{5},\phi_{6}\right)\in C\left(\left[-\tau ,0\right],{\mathbb{R}}_{+}^{6}\right).$$

The functions ϕ_*i*_ are assumed to be continuous, bounded, and non-negative on [−τ, 0]. To clarify the stochastic interpretation of the proposed model, we note that system (Equations 1–6) is a stochastic delay differential system because the population dynamics are perturbed by multiplicative Brownian noise terms. Therefore, the solution of the full model is a stochastic process rather than a deterministic trajectory. Consequently, the disease-free and endemic equilibria discussed in the following sections are understood as equilibria of the deterministic drift component of the stochastic system. The stochastic perturbations are then used to study sample-path fluctuations, stochastic extinction, persistence, and the modified threshold quantity ${ℛ}_{0}^{S}$.

## 4 Qualitative analysis

The qualitative analysis verifies positivity, boundedness, feasibility, and well-posedness of the stochastic delay hantavirus SIR–SIR model with noise, delays, and exponential survival effects.

### 4.1 Feasible region

Let


$$N_{H}\left(t\right)=S_{H}\left(t\right)+I_{H}\left(t\right)+R_{H}\left(t\right),\;N_{R}\left(t\right)=S_{R}\left(t\right)+I_{R}\left(t\right)+R_{R}\left(t\right),$$

where *N*_*H*_(*t*) and *N*_*R*_(*t*) denote the total human and rodent populations, respectively. Since all compartments represent biological populations, the natural state space of the model is defined by


$$\begin{array}{l} \Omega =\{\left(S_{H},I_{H},R_{H},S_{R},I_{R},R_{R}\right)\in {\mathbb{R}}_{+}^{6}:N_{H}\left(t\right)<\infty ,N_{R}\left(t\right)<\infty \}. \end{array}$$ (7)

Thus, the biologically feasible region is the non-negative cone ${\mathbb{R}}_{+}^{6}$, together with finite total human and rodent populations.

**Theorem 7** (Existence and uniqueness). For any vector-valued initial history function


X(θ)=ϕ(θ),  θ∈[-τ,0],

where


$$\phi =\left(\phi_{1},\phi_{2},\phi_{3},\phi_{4},\phi_{5},\phi_{6}\right)\in C\left(\left[-\tau ,0\right],{\mathbb{R}}_{+}^{6}\right),$$

the stochastic delay hantavirus model (Equations 1–6) has a unique local solution


$$X\left(t\right)=\left(S_{H}\left(t\right),I_{H}\left(t\right),R_{H}\left(t\right),S_{R}\left(t\right),I_{R}\left(t\right),R_{R}\left(t\right)\right)$$

for *t* ≥ 0.

*Proof*. The drift and diffusion coefficients of system (Equations 1–6) are locally Lipschitz continuous with respect to the present and delayed state variables. Therefore, by the standard existence and uniqueness theorem for stochastic delay differential equations (23), there exists a unique local solution for every continuous non-negative vector-valued initial history function


$$\phi \in C\left(\left[-\tau ,0\right],{\mathbb{R}}_{+}^{6}\right).$$

This proves the result.

**Theorem 8** (Non-negativity). *Let*


$$X\left(t\right)=\left(S_{H}\left(t\right),I_{H}\left(t\right),R_{H}\left(t\right),S_{R}\left(t\right),I_{R}\left(t\right),R_{R}\left(t\right)\right)$$

be the solution of system (Equations 1–6). If


$$X\left(\theta \right)\in {\mathbb{R}}_{+}^{6},\;\theta \in \left[-\tau ,0\right],$$

then


$$S_{H}\left(t\right)\geq 0,\;I_{H}\left(t\right)\geq 0,\;R_{H}\left(t\right)\geq 0,$$

$$S_{R}\left(t\right)\geq 0,\;I_{R}\left(t\right)\geq 0,\;R_{R}\left(t\right)\geq 0$$

for all *t* ≥ 0 almost surely.

*Proof*. The stochastic perturbations in the model are of multiplicative type, namely σ_*i*_*X*_*i*_(*t*)*dW*_*i*_(*t*), where *X*_*i*_(*t*) denotes the corresponding state variable. Thus, the diffusion term vanishes whenever the corresponding population component reaches zero. Moreover, all inflow terms are non-negative, whereas removal terms are proportional to the corresponding state variables.

For example, when *S*_*H*_(*t*) = 0, the drift term in the *S*_*H*_-equation becomes


$$\Gamma_{H}\geq 0,$$

and the diffusion term becomes


$$\sigma_{1}S_{H}\left(t\right)dW_{1}\left(t\right)=0.$$

Hence, the solution cannot cross from the non-negative region into the negative region through the boundary *S*_*H*_ = 0. Similar arguments apply to *I*_*H*_, *R*_*H*_, *S*_*R*_, *I*_*R*_, and *R*_*R*_. Therefore, all state variables remain non-negative for all *t* ≥ 0 almost surely.

**Theorem 9** (Stochastic boundedness). All non-negative solutions of system (Equations 1–6) are stochastically bounded. In particular, there exist positive constants *M*_*H*_ and *M*_*R*_ such that


$$\underset{t\to \infty }{lim sup}𝔼\left[N_{H}\left(t\right)\right]\leq M_{H},\;\underset{t\to \infty }{lim sup}𝔼\left[N_{R}\left(t\right)\right]\leq M_{R}.$$

*Proof*. Adding the first three equations of the stochastic delay system gives the total human population:


$$dN_{H}\left(t\right)=\left[\Gamma_{H}-\mu_{H}S_{H}\left(t\right)-\mu_{H}I_{H}\left(t\right)-\mu_{H}R_{H}\left(t\right)\right]dt$$

$$+\sigma_{1}S_{H}\left(t\right)dW_{1}\left(t\right)+\sigma_{2}I_{H}\left(t\right)dW_{2}\left(t\right)+\sigma_{3}R_{H}\left(t\right)dW_{3}\left(t\right).$$

Since


$$N_{H}\left(t\right)=S_{H}\left(t\right)+I_{H}\left(t\right)+R_{H}\left(t\right),$$

we obtain


$$dN_{H}\left(t\right)=\left[\Gamma_{H}-\mu_{H}N_{H}\left(t\right)\right]$$

$$dt+\sigma_{1}S_{H}\left(t\right)dW_{1}\left(t\right)+\sigma_{2}I_{H}\left(t\right)dW_{2}\left(t\right)+\sigma_{3}R_{H}\left(t\right)dW_{3}\left(t\right).$$

Taking expectation and using


$$𝔼\left[dW_{i}\left(t\right)\right]=0,$$

we have


$$\frac{d𝔼\left[N_{H}\left(t\right)\right]}{dt}=\Gamma_{H}-\mu_{H}𝔼\left[N_{H}\left(t\right)\right].$$

Solving this linear differential equation gives


$$𝔼\left[N_{H}\left(t\right)\right]=𝔼\left[N_{H}\left(0\right)\right]e^{-\mu_{H}t}+\frac{\Gamma_{H}}{\mu_{H}}\left(1-e^{-\mu_{H}t}\right).$$

Hence,


$$\underset{t\to \infty }{lim sup}𝔼\left[N_{H}\left(t\right)\right]\leq \frac{\Gamma_{H}}{\mu_{H}}.$$

Therefore, one may take


$$M_{H}=\frac{\Gamma_{H}}{\mu_{H}}.$$

Similarly, summing the rodent equations yields


$$dN_{R}\left(t\right)=\left[\Gamma_{R}-\mu_{R}N_{R}\left(t\right)\right]$$

$$dt+\sigma_{4}S_{R}\left(t\right)dW_{4}\left(t\right)+\sigma_{5}I_{R}\left(t\right)dW_{5}\left(t\right)+\sigma_{6}R_{R}\left(t\right)dW_{6}\left(t\right).$$

Taking expectation gives


$$\frac{d𝔼\left[N_{R}\left(t\right)\right]}{dt}=\Gamma_{R}-\mu_{R}𝔼\left[N_{R}\left(t\right)\right].$$

Solving this linear differential equation gives


$$𝔼\left[N_{R}\left(t\right)\right]=𝔼\left[N_{R}\left(0\right)\right]e^{-\mu_{R}t}+\frac{\Gamma_{R}}{\mu_{R}}\left(1-e^{-\mu_{R}t}\right).$$

Consequently,


$$\underset{t\to \infty }{lim sup}𝔼\left[N_{R}\left(t\right)\right]\leq \frac{\Gamma_{R}}{\mu_{R}}.$$

Hence, one may take


$$M_{R}=\frac{\Gamma_{R}}{\mu_{R}}.$$

Therefore, the human and rodent populations are bounded in expectation.

### 4.2 Disease-free equilibrium

The disease-free equilibrium (DFE) represents the state of the system in which hantavirus infection is absent from both the human and rodent populations. This compartmental structure is retained in the present stochastic delay formulation. At the disease-free state, there are no infected or recovered individuals in either population. Hence,


$$I_{H}=R_{H}=I_{R}=R_{R}=0.$$

Therefore, the disease-free equilibrium associated with the drift part of system (Equations 1–6) is given by


$$\begin{array}{l} {ℰ}_{0}=\left(S_{H}^{0},0,0,S_{R}^{0},0,0\right), \end{array}$$ (8)

where


$$S_{H}^{0}=\frac{\Gamma_{H}}{\mu_{H}},\;S_{R}^{0}=\frac{\Gamma_{R}}{\mu_{R}}.$$

Thus,


$$\begin{array}{l} {ℰ}_{0}=\left(\frac{\Gamma_{H}}{\mu_{H}},0,0,\frac{\Gamma_{R}}{\mu_{R}},0,0\right). \end{array}$$ (9)

### 4.3 Endemic equilibrium

The endemic equilibrium describes the long-term state in which hantavirus persists in the rodent population and consequently maintains infection in the human population. Since the full stochastic system contains Brownian perturbations, a constant point equilibrium is understood as the equilibrium of the deterministic drift part of the stochastic delay system. This approach is commonly used when analyzing stochastic epidemic systems around their deterministic skeleton.

Let the endemic equilibrium be denoted by


$${ℰ}^{*}=\left(S_{H}^{*},I_{H}^{*},R_{H}^{*},S_{R}^{*},I_{R}^{*},R_{R}^{*}\right),$$

where


$$S_{H}^{*}>0,\;I_{H}^{*}>0,\;R_{H}^{*}>0,\;S_{R}^{*}>0,\;I_{R}^{*}>0,\;R_{R}^{*}>0.$$

At equilibrium, the state variables are constant, and hence


$$X\left(t\right)=X\left(t-\tau_{H}\right)=X\left(t-\tau_{R}\right)=X^{*}.$$

For convenience, define the effective delayed transmission coefficients


$$\alpha_{H}=b\beta_{H}e^{-\eta_{H}\tau_{H}},\;\alpha_{R}=\beta_{R}e^{-\eta_{R}\tau_{R}}.$$

Here, α_*H*_ represents the effective rodent-to-human transmission rate after the human infection delay, while α_*R*_ represents the effective rodent-to-rodent transmission rate after the rodent transmission delay.

Setting the drift terms of system (Equations 1–6) equal to zero gives


$$\begin{array}{l} \Gamma_{H}-\alpha_{H}S_{H}^{*}I_{R}^{*}-\mu_{H}S_{H}^{*}=0, \end{array}$$ (10)

$$\begin{array}{l} \alpha_{H}S_{H}^{*}I_{R}^{*}-\left(\mu_{H}+\gamma_{H}\right)I_{H}^{*}=0, \end{array}$$ (11)

$$\begin{array}{l} \gamma_{H}I_{H}^{*}-\mu_{H}R_{H}^{*}=0, \end{array}$$ (12)

$$\begin{array}{l} \Gamma_{R}-\alpha_{R}S_{R}^{*}I_{R}^{*}-\mu_{R}S_{R}^{*}=0, \end{array}$$ (13)

$$\begin{array}{l} \alpha_{R}S_{R}^{*}I_{R}^{*}-\left(\mu_{R}+\gamma_{R}\right)I_{R}^{*}=0, \end{array}$$ (14)

$$\begin{array}{l} \gamma_{R}I_{R}^{*}-\mu_{R}R_{R}^{*}=0. \end{array}$$ (15)

From Equation 14, since $I_{R}^{*}>0$ at the endemic equilibrium, we obtain


$$\begin{array}{l} \alpha_{R}S_{R}^{*}=\mu_{R}+\gamma_{R}. \end{array}$$

Hence,


$$\begin{array}{l} \begin{array}{c} S_{R}^{*}=\frac{\mu_{R}+\gamma_{R}}{\alpha_{R}}=\frac{\mu_{R}+\gamma_{R}}{\beta_{R}e^{-\eta_{R}\tau_{R}}}. \end{array} \end{array}$$ (16)

Substituting Equation 16 into Equation 13 gives


$$\begin{array}{l} \Gamma_{R}-\left(\mu_{R}+\gamma_{R}\right)I_{R}^{*}-\mu_{R}S_{R}^{*}=0. \end{array}$$ (17)

Therefore,


$$\begin{array}{l} \begin{array}{c} I_{R}^{*}=\frac{\Gamma_{R}}{\mu_{R}+\gamma_{R}}-\frac{\mu_{R}}{\alpha_{R}}=\frac{\Gamma_{R}}{\mu_{R}+\gamma_{R}}-\frac{\mu_{R}}{\beta_{R}e^{-\eta_{R}\tau_{R}}}. \end{array} \end{array}$$ (18)

From Equation 15, the recovered rodent population is


$$\begin{array}{l} \begin{array}{c} R_{R}^{*}=\frac{\gamma_{R}}{\mu_{R}}I_{R}^{*}. \end{array} \end{array}$$ (19)

Next, from Equation 10, the susceptible human population satisfies


$$\begin{array}{l} S_{H}^{*}\left(\mu_{H}+\alpha_{H}I_{R}^{*}\right)=\Gamma_{H}. \end{array}$$ (20)

Thus,


$$\begin{array}{l} \begin{array}{c} S_{H}^{*}=\frac{\Gamma_{H}}{\mu_{H}+\alpha_{H}I_{R}^{*}}=\frac{\Gamma_{H}}{\mu_{H}+b\beta_{H}e^{-\eta_{H}\tau_{H}}I_{R}^{*}}. \end{array} \end{array}$$ (21)

From Equation 11, we obtain


$$\begin{array}{l} \begin{array}{c} I_{H}^{*}=\frac{\alpha_{H}S_{H}^{*}I_{R}^{*}}{\mu_{H}+\gamma_{H}}=\frac{b\beta_{H}e^{-\eta_{H}\tau_{H}}S_{H}^{*}I_{R}^{*}}{\mu_{H}+\gamma_{H}}. \end{array} \end{array}$$ (22)

Finally, from Equation 12, the recovered human population is


$$\begin{array}{l} \begin{array}{c} R_{H}^{*}=\frac{\gamma_{H}}{\mu_{H}}I_{H}^{*}. \end{array} \end{array}$$ (23)

Hence, the endemic equilibrium of the deterministic drift component of the stochastic delay hantavirus model is


$$\begin{array}{l} \begin{array}{c} {ℰ}^{*}=\left(S_{H}^{*},I_{H}^{*},R_{H}^{*},S_{R}^{*},I_{R}^{*},R_{R}^{*}\right), \end{array} \end{array}$$ (24)

where $S_{H}^{*}$, $I_{H}^{*}$, $R_{H}^{*}$, $S_{R}^{*}$, $I_{R}^{*}$, and $R_{R}^{*}$ are given by Equations 21–19. The endemic equilibrium exists only if


$$I_{R}^{*}>0.$$

Using Equations 18, this condition becomes


$$\frac{\Gamma_{R}}{\mu_{R}+\gamma_{R}}>\frac{\mu_{R}}{\alpha_{R}}.$$

Equivalently,


$$\frac{\alpha_{R}\Gamma_{R}}{\mu_{R}\left(\mu_{R}+\gamma_{R}\right)}>1.$$

Therefore, the delayed basic reproduction number is defined as


$$\begin{array}{l} \begin{array}{c} {ℛ}_{0}^{d}=\frac{\beta_{R}e^{-\eta_{R}\tau_{R}}\Gamma_{R}}{\mu_{R}\left(\mu_{R}+\gamma_{R}\right)}. \end{array} \end{array}$$ (25)

Thus, the endemic equilibrium exists whenever ${ℛ}_{0}^{d}>1$.

### 4.4 Basic reproduction number

The basic reproduction number, denoted by ℛ_0_, represents the expected number of secondary infected rodents generated by one infected rodent introduced into a fully susceptible rodent population. For the hantavirus model considered here, infection is transmitted among rodents and from infected rodents to humans, while human-to-human and human-to-rodent transmission are neglected. Therefore, the persistence threshold is determined by the rodent transmission cycle.

We compute the basic reproduction number using the next-generation matrix method (24, 25). To avoid notational ambiguity, the infected subsystem is denoted by the vector


$$Y_{I}=\left(\begin{array}{c} I_{H}\\ I_{R} \end{array}\right).$$

This notation distinguishes the infected subsystem from the full six-dimensional state vector *X*(*t*) = (*S*_*H*_(*t*), *I*_*H*_(*t*), *R*_*H*_(*t*), *S*_*R*_(*t*), *I*_*R*_(*t*), *R*_*R*_(*t*)).

Define the effective delayed transmission coefficients


$$\alpha_{H}=b\beta_{H}e^{-\eta_{H}\tau_{H}},\;\alpha_{R}=\beta_{R}e^{-\eta_{R}\tau_{R}}.$$

Here, α_*H*_ is the effective delayed transmission rate from infected rodents to humans, whereas α_*R*_ is the effective delayed transmission rate among rodents.

The vector of new infection terms is denoted by


$${ℱ}_{I}\left(Y_{I}\right)=\left(\begin{array}{c} \alpha_{H}S_{H}I_{R}\\ \alpha_{R}S_{R}I_{R} \end{array}\right),$$

and the vector of transition terms is denoted by


$${𝒱}_{I}\left(Y_{I}\right)=\left(\begin{array}{c} \left(\mu_{H}+\gamma_{H}\right)I_{H}\\ \left(\mu_{R}+\gamma_{R}\right)I_{R} \end{array}\right).$$

At the disease-free equilibrium


$${ℰ}_{0}=\left(\frac{\Gamma_{H}}{\mu_{H}},0,0,\frac{\Gamma_{R}}{\mu_{R}},0,0\right),$$

we have


$$S_{H}^{0}=\frac{\Gamma_{H}}{\mu_{H}},\;S_{R}^{0}=\frac{\Gamma_{R}}{\mu_{R}}.$$

The Jacobian matrices of ℱ_*I*_ and 𝒱_*I*_ with respect to the infected variables (*I*_*H*_, *I*_*R*_) at ℰ_0_ are denoted by *F*_*J*_ and *V*_*J*_, respectively, and are given by


$$F_{J}=\left(\begin{array}{cc} 0 & \alpha_{H}S_{H}^{0}\\ 0 & \alpha_{R}S_{R}^{0} \end{array}\right),\;V_{J}=\left(\begin{array}{cc} \mu_{H}+\gamma_{H} & 0\\ 0 & \mu_{R}+\gamma_{R} \end{array}\right).$$

Thus,


$$V_{J}^{-1}=\left(\begin{array}{cc} \frac{1}{\mu_{H}+\gamma_{H}} & 0\\ 0 & \frac{1}{\mu_{R}+\gamma_{R}} \end{array}\right).$$

The next-generation matrix is


$$𝒦=F_{J}V_{J}^{-1},$$

where


$$𝒦=\left(\begin{array}{cc} 0 & \frac{\alpha_{H}S_{H}^{0}}{\mu_{R}+\gamma_{R}}\\ 0 & \frac{\alpha_{R}S_{R}^{0}}{\mu_{R}+\gamma_{R}} \end{array}\right).$$

The delayed basic reproduction number is the spectral radius of 𝒦:


$${ℛ}_{0}^{d}=\rho \left(𝒦\right).$$

Since 𝒦 is an upper triangular matrix, its eigenvalues are


$$\lambda_{1}=0,\;\lambda_{2}=\frac{\alpha_{R}S_{R}^{0}}{\mu_{R}+\gamma_{R}}.$$

Therefore,


$$\begin{array}{l} \begin{array}{c} {ℛ}_{0}^{d}=\frac{\alpha_{R}S_{R}^{0}}{\mu_{R}+\gamma_{R}}. \end{array} \end{array}$$ (26)

Using


$$S_{R}^{0}=\frac{\Gamma_{R}}{\mu_{R}}\text{ and }\alpha_{R}=\beta_{R}e^{-\eta_{R}\tau_{R}},$$

we obtain


$$\begin{array}{l} \begin{array}{c} {ℛ}_{0}^{d}=\frac{\beta_{R}e^{-\eta_{R}\tau_{R}}\Gamma_{R}}{\mu_{R}\left(\mu_{R}+\gamma_{R}\right)}. \end{array} \end{array}$$ (27)

### 4.5 Stochastic basic reproduction number

In this subsection, we derive a stochastic threshold for the delayed hantavirus model under environmental noise. Therefore, the stochastic persistence threshold is mainly determined by the infected rodent equation. Near the disease-free equilibrium


$${ℰ}_{0}=\left(\frac{\Gamma_{H}}{\mu_{H}},0,0,\frac{\Gamma_{R}}{\mu_{R}},0,0\right),$$

the infected subsystem is given by


$$X\left(t\right)=\left(\begin{array}{c} I_{H}\left(t\right)\\ I_{R}\left(t\right) \end{array}\right).$$

Linearizing system (Equations 1–6) around ℰ_0_, we obtain


$$\begin{array}{r} \begin{array}{r} dI_{H}\left(t\right)=\left[\alpha_{H}S_{H}^{0}I_{R}\left(t-\tau_{H}\right)-\left(\mu_{H}+\gamma_{H}\right)I_{H}\left(t\right)\right]\\ dt+\sigma_{2}I_{H}\left(t\right)dW_{2}\left(t\right),\\ dI_{R}\left(t\right)=\left[\alpha_{R}S_{R}^{0}I_{R}\left(t-\tau_{R}\right)-\left(\mu_{R}+\gamma_{R}\right)I_{R}\left(t\right)\right]\\ dt+\sigma_{5}I_{R}\left(t\right)dW_{5}\left(t\right), \end{array} \end{array}$$ (28)

where


$$S_{H}^{0}=\frac{\Gamma_{H}}{\mu_{H}},\;S_{R}^{0}=\frac{\Gamma_{R}}{\mu_{R}},$$

and


$$\alpha_{H}=b\beta_{H}e^{-\eta_{H}\tau_{H}},\;\alpha_{R}=\beta_{R}e^{-\eta_{R}\tau_{R}}.$$

Here, α_*H*_ denotes the effective delayed transmission rate from infected rodents to humans, whereas α_*R*_ denotes the effective delayed transmission rate among rodents.

Since humans are dead-end hosts in this model, the equation for *I*_*H*_(*t*) is driven by *I*_*R*_(*t*) but does not feed infection back into the rodent population. Hence, the stochastic threshold is determined by the infected rodent equation:


$$\begin{array}{l} dI_{R}\left(t\right)=\left[\alpha_{R}S_{R}^{0}I_{R}\left(t-\tau_{R}\right)-\left(\mu_{R}+\gamma_{R}\right)I_{R}\left(t\right)\right]dt+\sigma_{5}I_{R}\left(t\right)dW_{5}\left(t\right). \end{array}$$ (29)

For the corresponding non-delayed stochastic linear equation, applying Itô's formula to ln *I*_*R*_(*t*) gives the almost-sure growth rate


$$\alpha_{R}S_{R}^{0}-\left(\mu_{R}+\gamma_{R}\right)-\frac{\sigma_{5}^{2}}{2}.$$

Thus, environmental noise reduces the almost-sure growth rate of infected rodents by $\sigma_{5}^{2}/2$. This leads to the following stochastic basic reproduction number:


$$\begin{array}{l} \begin{array}{c} {ℛ}_{0}^{S}=\frac{\alpha_{R}S_{R}^{0}}{\left(\mu_{R}+\gamma_{R}\right)+\sigma_{5}^{2}2}. \end{array} \end{array}$$ (30)

Substituting


$$\alpha_{R}=\beta_{R}e^{-\eta_{R}\tau_{R}},\;S_{R}^{0}=\frac{\Gamma_{R}}{\mu_{R}},$$

we obtain


$$\begin{array}{l} \begin{array}{c} {ℛ}_{0}^{S}=\frac{\beta_{R}e^{-\eta_{R}\tau_{R}}\Gamma_{R}}{\mu_{R}\left[\left(\mu_{R}+\gamma_{R}\right)+\sigma_{5}^{2}2\right]}. \end{array} \end{array}$$ (31)

The threshold condition is therefore given by


$${ℛ}_{0}^{S}<1.$$

If this inequality holds, then infected rodents tend to die out almost surely, and the infection cannot be sustained in the rodent population. Since human infection is generated by infected rodents, extinction of *I*_*R*_(*t*) also leads to extinction of spillover infection in humans.

**Remark 10**. In the absence of stochastic perturbation in the infected rodent class, that is, when σ_5_ = 0, the stochastic threshold reduces to the delayed deterministic reproduction number


$${ℛ}_{0}^{d}=\frac{\beta_{R}e^{-\eta_{R}\tau_{R}}\Gamma_{R}}{\mu_{R}\left(\mu_{R}+\gamma_{R}\right)}.$$

Thus,


$${ℛ}_{0}^{S}\leq {ℛ}_{0}^{d},$$

which shows that multiplicative noise in the infected rodent compartment decreases the almost-sure persistence potential of hantavirus.

**Remark 11**. If the threshold is derived from mean-square stability rather than almost-sure logarithmic growth, then the correction has the opposite sign. Applying Itô's formula to $I_{R}^{2}\left(t\right)$ gives


$$d\left(I_{R}^{2}\right)=2I_{R}dI_{R}+{\left(dI_{R}\right)}^{2}.$$

For the non-delayed linearized infected rodent equation, this yields the second-moment growth coefficient


$$2\left(\alpha_{R}S_{R}^{0}-\left(\mu_{R}+\gamma_{R}\right)\right)+\sigma_{5}^{2}.$$

Hence, the corresponding mean-square threshold is


$${ℛ}_{0,MS}^{S}=\frac{\alpha_{R}S_{R}^{0}}{\left(\mu_{R}+\gamma_{R}\right)-\sigma_{5}^{2}2},\;\mu_{R}+\gamma_{R}>\frac{\sigma_{5}^{2}}{2}.$$

This quantity is used for mean-square extinction, whereas Equation 31 is used for almost-sure extinction.

### 4.6 Extinction and persistence

In this subsection, we establish sufficient conditions for the extinction and persistence of hantavirus infection in the stochastic delay model. The model assumes that transmission is maintained within the rodent population and that humans acquire infection through contact with infected rodents or contaminated environments, while human-to-human and human-to-rodent transmission are neglected (26). Therefore, the long-term extinction or persistence of hantavirus is mainly governed by the infected rodent population. In particular, the rodent transmission cycle determines the threshold condition for disease persistence in the proposed framework.

Let


$$\alpha_{H}=b\beta_{H}e^{-\eta_{H}\tau_{H}},\;\alpha_{R}=\beta_{R}e^{-\eta_{R}\tau_{R}}.$$

Near the disease-free equilibrium


$${ℰ}_{0}=\left(\frac{\Gamma_{H}}{\mu_{H}},0,0,\frac{\Gamma_{R}}{\mu_{R}},0,0\right),$$

the infected rodent equation is governed by


$$\begin{array}{l} dI_{R}\left(t\right)=\left[\alpha_{R}S_{R}^{0}I_{R}\left(t-\tau_{R}\right)-\left(\mu_{R}+\gamma_{R}\right)I_{R}\left(t\right)\right]dt+\sigma_{5}I_{R}\left(t\right)dW_{5}\left(t\right), \end{array}$$ (32)

where


$$S_{R}^{0}=\frac{\Gamma_{R}}{\mu_{R}}.$$

**Theorem 12**. *Assume that*


$${ℛ}_{0}^{S}=\frac{\beta_{R}e^{-\eta_{R}\tau_{R}}\Gamma_{R}}{\mu_{R}\left[\left(\mu_{R}+\gamma_{R}\right)+\sigma_{5}^{2}2\right]}<1.$$

Then the infected rodent population becomes extinct almost surely, that is,


$$\underset{t\to \infty }{\lim}I_{R}\left(t\right)=0\text{ a.s.}$$

Consequently, the spillover infection in humans also disappears in the sense that


$$\underset{t\to \infty }{\lim}I_{H}\left(t\right)=0\text{ a.s.}$$

*Proof*. Since the infected human class is generated by infected rodents but does not feed infection back into the rodent population, it is sufficient to analyze the asymptotic behavior of *I*_*R*_(*t*). Consider the logarithmic Lyapunov function


$$V\left(t\right)=\ln\;I_{R}\left(t\right).$$

Applying Itô's formula to Equation 32 gives


$$d\ln\;I_{R}\left(t\right)=\left[\alpha_{R}S_{R}^{0}\frac{I_{R}\left(t-\tau_{R}\right)}{I_{R}\left(t\right)}-\left(\mu_{R}+\gamma_{R}\right)-\frac{\sigma_{5}^{2}}{2}\right]dt+\sigma_{5}dW_{5}\left(t\right).$$

For the infection to grow near the disease-free state, the delayed production term must exceed the total stochastic removal rate. Thus, a sufficient condition for negative logarithmic growth is


$$\alpha_{R}S_{R}^{0}<\left(\mu_{R}+\gamma_{R}\right)+\frac{\sigma_{5}^{2}}{2}.$$

Equivalently,


$$\frac{\alpha_{R}S_{R}^{0}}{\left(\mu_{R}+\gamma_{R}\right)+\frac{\sigma_{5}^{2}}{2}.}<1.$$

Using


$$\alpha_{R}=\beta_{R}e^{-\eta_{R}\tau_{R}},\;S_{R}^{0}=\frac{\Gamma_{R}}{\mu_{R}},$$

this condition becomes


$${ℛ}_{0}^{S}=\frac{\beta_{R}e^{-\eta_{R}\tau_{R}}\Gamma_{R}}{\mu_{R}\left[\left(\mu_{R}+\gamma_{R}\right)+\sigma_{5}^{2}2\right]}<1.$$

Integrating the logarithmic equation from 0 to *t* and dividing by *t* yields


$$\frac{\ln\;I_{R}\left(t\right)-\ln\;I_{R}\left(0\right)}{t}\leq -\kappa +\frac{\sigma_{5}W_{5}\left(t\right)}{t},$$

for some κ > 0. By the strong law of large numbers for Brownian motion,


$$\underset{t\to \infty }{\lim}\frac{W_{5}\left(t\right)}{t}=0$$

Therefore,


$$\underset{t\to \infty }{lim sup}\frac{1}{t}\ln\;I_{R}\left(t\right)\leq -\kappa <0\text{ a.s.}$$

This implies


$$\underset{t\to \infty }{\lim}I_{R}\left(t\right)=0$$

Since the infected human equation is


$$dI_{H}\left(t\right)=\left[\alpha_{H}S_{H}\left(t-\tau_{H}\right)I_{R}\left(t-\tau_{H}\right)-\left(\mu_{H}+\gamma_{H}\right)I_{H}\left(t\right)\right]$$

$$dt+\sigma_{2}I_{H}\left(t\right)dW_{2}\left(t\right),$$

the disappearance of *I*_*R*_(*t*) removes the source term of human infection. Hence,


$$\underset{t\to \infty }{\lim}I_{H}\left(t\right)=0\text{ a.s.}$$

This completes the proof.

**Theorem 13**. *Assume that ${ℛ}_{0}^{S}<1$, then the infected rodent population is exponentially stable in the mean-square sense. In particular,*


$$\underset{t\to \infty }{\lim}𝔼\left[I_{R}^{2}\left(t\right)\right]=0.$$

*Consequently,*


$$\underset{t\to \infty }{\lim}𝔼\left[I_{H}^{2}\left(t\right)\right]=0.$$

*Proof*. Define the Lyapunov function


$$V\left(t\right)=I_{R}^{2}\left(t\right).$$

Applying Itô's formula gives


$$dV\left(t\right)=2I_{R}\left(t\right)dI_{R}\left(t\right)+{\left(dI_{R}\left(t\right)\right)}^{2}.$$

Using Equation 32, we obtain


$$\begin{array}{r} dI_{R}^{2}\left(t\right)=[2\alpha_{R}S_{R}^{0}I_{R}\left(t\right)I_{R}\left(t-\tau_{R}\right)\\ -2\left(\mu_{R}+\gamma_{R}\right)I_{R}^{2}\left(t\right)+\sigma_{5}^{2}I_{R}^{2}\left(t\right)]dt\\ +2\sigma_{5}I_{R}^{2}\left(t\right)dW_{5}\left(t\right). \end{array}$$

Taking expectation gives


$$\frac{d}{dt}𝔼\left[I_{R}^{2}\left(t\right)\right]=2\alpha_{R}S_{R}^{0}𝔼\left[I_{R}\left(t\right)I_{R}\left(t-\tau_{R}\right)\right]-$$

$$\left[2\left(\mu_{R}+\gamma_{R}\right)-\sigma_{5}^{2}\right]𝔼\left[I_{R}^{2}\left(t\right)\right].$$

Using Young's inequality,


$$2xy\leq x^{2}+y^{2},$$

we have


$$2𝔼\left[I_{R}\left(t\right)I_{R}\left(t-\tau_{R}\right)\right]\leq 𝔼\left[I_{R}^{2}\left(t\right)\right]+𝔼\left[I_{R}^{2}\left(t-\tau_{R}\right)\right].$$

Thus,


$$\frac{d}{dt}𝔼\left[I_{R}^{2}\left(t\right)\right]\leq \alpha_{R}S_{R}^{0}\left[𝔼\left[I_{R}^{2}\left(t\right)\right]+𝔼\left[I_{R}^{2}\left(t-\tau_{R}\right)\right]\right]-$$

$$\left[2\left(\mu_{R}+\gamma_{R}\right)-\sigma_{5}^{2}\right]𝔼\left[I_{R}^{2}\left(t\right)\right].$$

A sufficient condition for exponential decay of the second moment is


$$2\alpha_{R}S_{R}^{0}<2\left(\mu_{R}+\gamma_{R}\right)-\sigma_{5}^{2},$$

or equivalently,


$$\alpha_{R}S_{R}^{0}<\left(\mu_{R}+\gamma_{R}\right)-\frac{\sigma_{5}^{2}}{2}.$$

Therefore,


$${ℛ}_{0,MS}^{S}=\frac{\alpha_{R}S_{R}^{0}}{\left(\mu_{R}+\gamma_{R}\right)-\frac{\sigma_{5}^{2}}{2}}<1.$$

Under this condition,


$$\underset{t\to \infty }{\lim}𝔼\left[I_{R}^{2}\left(t\right)\right]=0.$$

Since infected humans are produced only through contact with infected rodents, the extinction of *I*_*R*_(*t*) also removes the source of human infection. Hence,


$$\underset{t\to \infty }{\lim}𝔼\left[I_{H}^{2}\left(t\right)\right]=0.$$

This proves the result.

**Theorem 14**. *Assume that ${ℛ}_{0}^{S}>1$, then hantavirus infection persists in the rodent population in the mean sense. That is, there exists a constant η > 0 such that*


$$\underset{t\to \infty }{\mathrm{liminf}}\frac{1}{t}\int_{0}^{t}𝔼\left[I_{R}\left(s\right)\right]ds\geq \eta .$$

*Proof*. The infected rodent equation is


$$dI_{R}\left(t\right)=\left[\alpha_{R}S_{R}\left(t-\tau_{R}\right)I_{R}\left(t-\tau_{R}\right)-\left(\mu_{R}+\gamma_{R}\right)I_{R}\left(t\right)\right]-$$

$$dt+\sigma_{5}I_{R}\left(t\right)dW_{5}\left(t\right).$$

Near the disease-free state, *S*_*R*_(*t*) remains close to


$$S_{R}^{0}=\frac{\Gamma_{R}}{\mu_{R}}.$$

Applying Itô's formula to ln *I*_*R*_(*t*) gives


$$d\ln\;I_{R}\left(t\right)=\left[\alpha_{R}S_{R}\left(t-\tau_{R}\right)\frac{I_{R}\left(t-\tau_{R}\right)}{I_{R}\left(t\right)}-\left(\mu_{R}+\gamma_{R}\right)-\frac{\sigma_{5}^{2}}{2}\right]-$$

$$dt+\sigma_{5}dW_{5}\left(t\right).$$

If


$${ℛ}_{0}^{S}>1,$$

then


$$\alpha_{R}S_{R}^{0}>\left(\mu_{R}+\gamma_{R}\right)+\frac{\sigma_{5}^{2}}{2}.$$

Hence, in a neighborhood of the disease-free state, the logarithmic growth rate of *I*_*R*_(*t*) is positive. Therefore, the infection cannot remain arbitrarily close to zero for all large time. Consequently, there exists a constant η > 0 such that


$$\underset{t\to \infty }{\mathrm{liminf}}\frac{1}{t}\int_{0}^{t}𝔼\left[I_{R}\left(s\right)\right]ds\geq \eta .$$

Since the infected human equation contains the source term


$$\alpha_{H}S_{H}\left(t-\tau_{H}\right)I_{R}\left(t-\tau_{H}\right),$$

a positive long-term mean of *I*_*R*_(*t*) implies a positive long-term mean input into the human infected class whenever


$$\alpha_{H}>0.$$

Thus, spillover infection in humans is maintained in the mean whenever the rodent infection persists and rodent-to-human transmission is active.

## 5 Real data analysis

This section presents reported hantavirus statistics from February 2026 to May 2026 and connects them with the stochastic delay hantavirus model. The available public-health reports do not provide a complete month-by-month surveillance dataset. Therefore, the values used here are treated as reported monthly update points rather than exact monthly incidence counts (see Table 2).

**Table 2:** Reported hantavirus update points from February to May 2026.

Month	Region / setting	Reported cases	Reported deaths
February 2026	Argentina, since start of 2026	19	Not reported
March 2026	Argentina, 1 January–28 March	32	8
April 2026	Argentina, 2025/2026 season	101	32
May 2026	Multi-country cruise-ship cluster	8	3

In February 2026, Argentina reported 19 hantavirus cases since the beginning of the year. By March 2026, 32 confirmed cases and eight deaths had been reported in Argentina between 1 January and 28 March 2026. In April 2026, Argentina reported 101 confirmed cases and 32 deaths during the 2025/2026 hantavirus season. In May 2026, WHO reported a multi-country hantavirus cluster linked to cruise-ship travel, with eight cases and three deaths as of 8 May 2026 (27, 28).

For the model-based analysis, we use the parameter values adopted in the deterministic hantavirus model:


$$b=0.1,\;\beta_{H}=0.0015,\;\Gamma_{H}=0.25,\;\gamma_{H}=\frac{1}{200},\;$$

$$\mu_{H}=\frac{1}{65\times 365},$$

$$\beta_{R}=0.002,\;\Gamma_{R}=0.25,\;\gamma_{R}=0.0075,\;\mu_{R}=0.007.$$

The stochastic delay reproduction number used for graphical analysis is


$${ℛ}_{0}^{S}=\frac{\beta_{R}e^{-\eta_{R}\tau_{R}}\Gamma_{R}}{\mu_{R}\left[\left(\mu_{R}+\gamma_{R}\right)+\frac{\sigma_{5}^{2}}{2}\right]}.$$

This expression shows that rodent-to-rodent transmission β_*R*_ increases the disease persistence potential, whereas the exponential delay factor $e^{-\eta_{R}\tau_{R}}$ and infected rodent noise intensity σ_5_ reduce the stochastic reproduction number.

**Figure 1:**
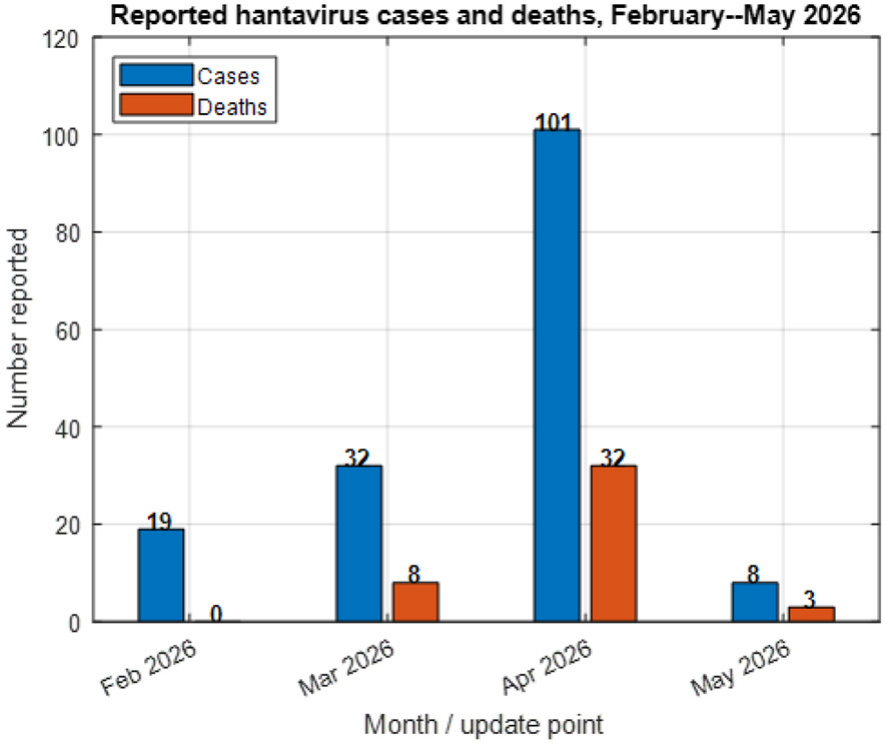
Reported hantavirus case update points from February to May 2026.

**Figure 2:**
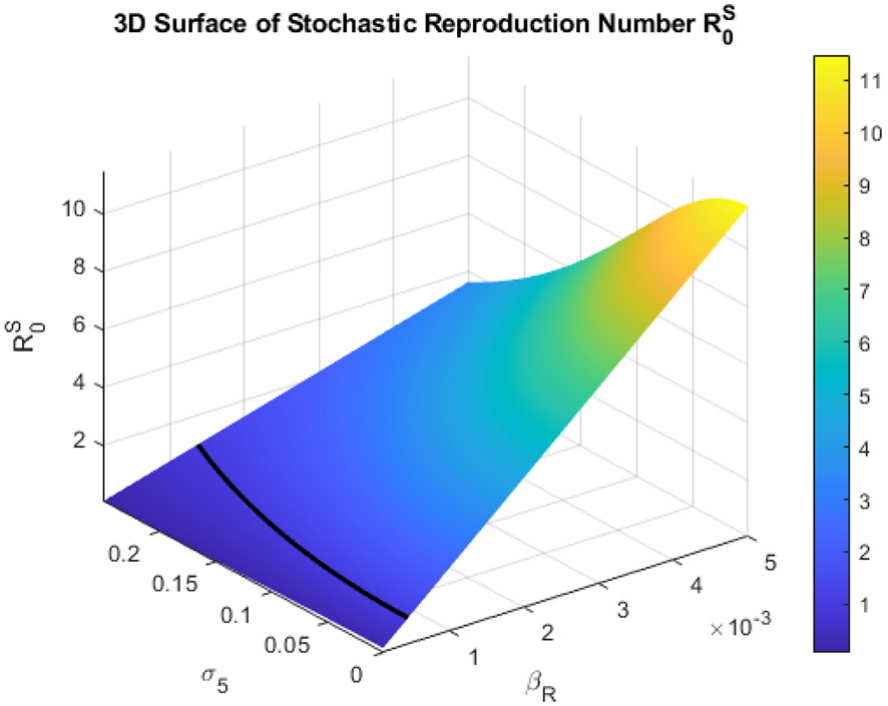
Three-dimensional surface of the stochastic reproduction number $R_{0}^{S}$ with respect to the rodent transmission rate β_*R*_ and infected-rodent noise intensity σ_5_. The black line represents the threshold contour $R_{0}^{S}=1$, which separates the extinction and persistence regions.

Figure 1 shows the reported hantavirus update points from February to May 2026. The reported cases increased from 19 in February to 32 in March, with the highest value of 101 cases reported in April. The April value represents a broader 2025/2026 seasonal update, so it should not be interpreted as cases occurring only in April. The May value of eight cases refers to a separate cruise-ship-associated cluster and is not directly comparable with the Argentina-based reports. Figure 2 shows the effect of the rodent transmission rate β_*R*_ and the infected-rodent noise intensity σ_5_ on the stochastic reproduction number ${ℛ}_{0}^{S}$. The surface increases as β_*R*_ increases, indicating that stronger rodent-to-rodent transmission promotes hantavirus persistence. In contrast, the surface decreases as σ_5_ increases because stochastic fluctuations reduce the effective persistence potential of infected rodents. The threshold contour ${ℛ}_{0}^{S}=1$ separates the extinction and persistence regions. When ${ℛ}_{0}^{S}<1$, the infection tends to die out; when ${ℛ}_{0}^{S}>1$, the infection may persist in the rodent population and continue causing spillover infections in humans. Therefore, the graphical results support the importance of reducing rodent transmission through sanitation, rodent control, and reduced exposure to contaminated environments.

## 6 Numerical scheme

Numerical methods are required to approximate solutions of stochastic delay differential equations when closed-form solutions are not available. For the stochastic delay hantavirus model, numerical simulation is useful for examining the effects of environmental noise, transmission delay, and exponential delay-survival factors on disease extinction or persistence. In this section, we present the pseudocode of the stochastic Euler–Maruyama and stochastic Runge–Kutta methods (see Algorithms 1 and 2), and then construct the stochastic nonstandard finite difference scheme for the proposed model (23, 29).

Algorithm 1 Stochastic Euler method. 
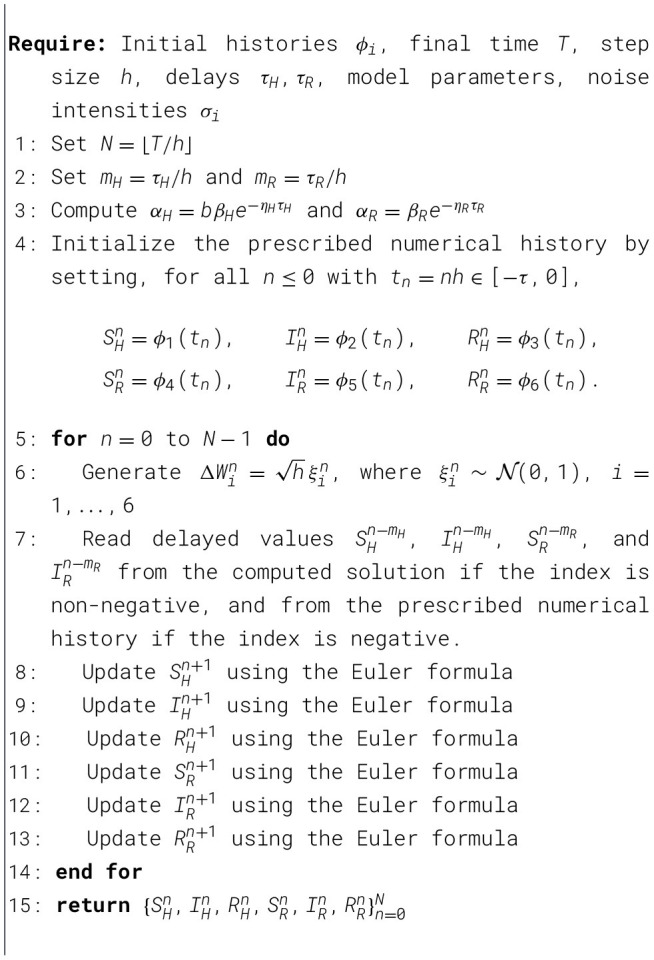

Algorithm 2 Stochastic Runge–Kutta method. 
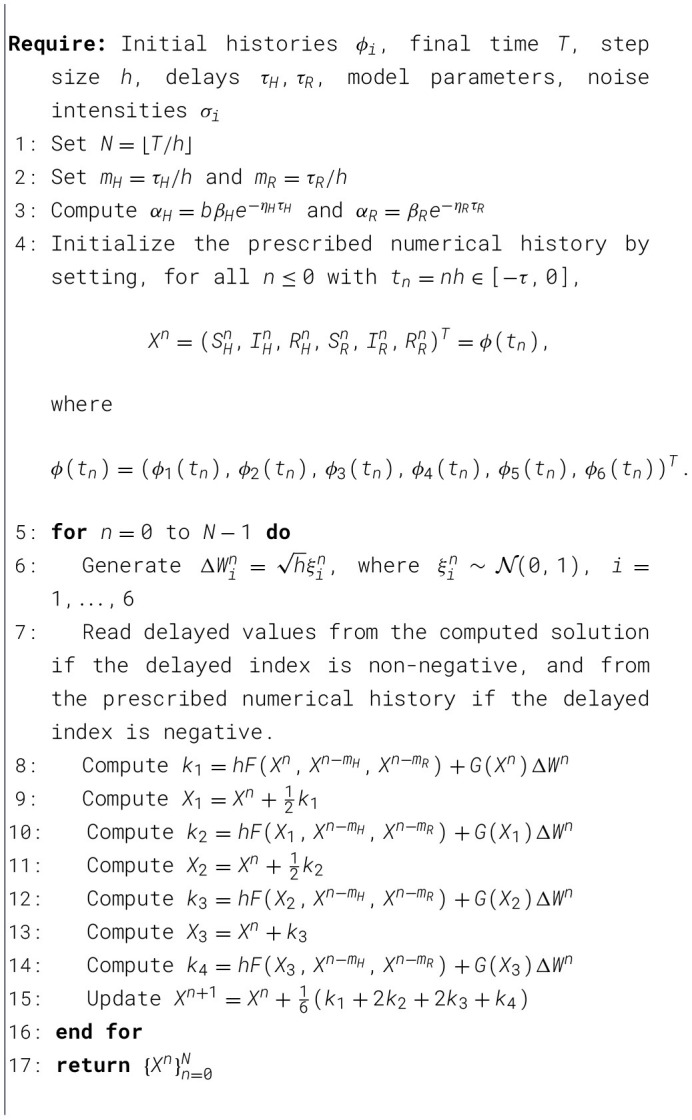

### 6.1 Pseudocode for stochastic Euler method

Before presenting the numerical algorithm, we clarify the indexing convention used for the delay terms. Let *t*_*n*_ = *nh* for *n* ∈ ℤ and let τ = max{τ_*H*_, τ_*R*_}. We assume that the delays are integer multiples of the step size, namely τ_*H*_ = *m*_*H*_*h* and τ_*R*_ = *m*_*R*_*h*, where *m*_*H*_, *m*_*R*_ ∈ ℕ_0_. The negative indices correspond to the prescribed history interval [−τ, 0]. Thus, for *n* ≤ 0, the values of the numerical solution are not computed by the recurrence scheme but are assigned directly from the initial history functions. If a delayed index such as *n* − *m*_*H*_ or *n* − *m*_*R*_ is negative during the time stepping, the corresponding value is read from this prescribed numerical history.

### 6.2 Pseudocode for stochastic Runge–Kutta method

The same delay-indexing convention is used for the stochastic Runge–Kutta method. Negative indices *n* ≤ 0 represent values prescribed by the initial history functions on the interval [−τ, 0]. During the computation, delayed values with negative indices are taken from this numerical history, while delayed values with non-negative indices are taken from the previously computed solution.

### 6.3 Stochastic non-standard finite difference scheme

We construct a stochastic NSFD scheme for the stochastic delay hantavirus model (Equations 1–6. The purpose of this scheme is to approximate the solution while preserving the biological feasibility of the human and rodent compartments


$$\left(S_{H},I_{H},R_{H},S_{R},I_{R},R_{R}\right).$$

Let *h* > 0 be the time step and define


$$t_{n}=nh,\;n=0,1,2,\ldots .$$

Assume that the delays satisfy


$$\tau_{H}=m_{H}h,\;\tau_{R}=m_{R}h,$$

where *m*_*H*_ and *m*_*R*_ are non-negative integers. Let


$$\Delta W_{i}^{n}\sim 𝒩\left(0,h\right),\;i=1,2,\ldots ,6,$$

be independent Brownian increments.

For simplicity, define the effective delayed transmission coefficients


$$\alpha_{H}=b\beta_{H}e^{-\eta_{H}\tau_{H}},\;\alpha_{R}=\beta_{R}e^{-\eta_{R}\tau_{R}}.$$

The stochastic NSFD scheme is given by


$$\begin{array}{l} S_{H}^{n+1}=\frac{S_{H}^{n}+h\Gamma_{H}+\sigma_{1}S_{H}^{n}\Delta W_{1}^{n}}{1+h\left(\mu_{H}+\alpha_{H}I_{R}^{n-m_{H}}\right)}, \end{array}$$ (33)

$$\begin{array}{l} I_{H}^{n+1}=\frac{I_{H}^{n}+h\alpha_{H}S_{H}^{n-m_{H}}I_{R}^{n-m_{H}}+\sigma_{2}I_{H}^{n}\Delta W_{2}^{n}}{1+h\left(\mu_{H}+\gamma_{H}\right)}, \end{array}$$ (34)

$$\begin{array}{l} R_{H}^{n+1}=\frac{R_{H}^{n}+h\gamma_{H}I_{H}^{n}+\sigma_{3}R_{H}^{n}\Delta W_{3}^{n}}{1+h\mu_{H}}, \end{array}$$ (35)

$$\begin{array}{l} S_{R}^{n+1}=\frac{S_{R}^{n}+h\Gamma_{R}+\sigma_{4}S_{R}^{n}\Delta W_{4}^{n}}{1+h\left(\mu_{R}+\alpha_{R}I_{R}^{n-m_{R}}\right)}, \end{array}$$ (36)

$$\begin{array}{l} I_{R}^{n+1}=\frac{I_{R}^{n}+h\alpha_{R}S_{R}^{n-m_{R}}I_{R}^{n-m_{R}}+\sigma_{5}I_{R}^{n}\Delta W_{5}^{n}}{1+h\left(\mu_{R}+\gamma_{R}\right)}, \end{array}$$ (37)

$$\begin{array}{l} R_{R}^{n+1}=\frac{R_{R}^{n}+h\gamma_{R}I_{R}^{n}+\sigma_{6}R_{R}^{n}\Delta W_{6}^{n}}{1+h\mu_{R}}. \end{array}$$ (38)

For *n* − *m*_*H*_ < 0 or *n* − *m*_*R*_ < 0, the delayed values are taken from the prescribed initial history functions:


$$S_{H}^{n}=\phi_{1}\left(t_{n}\right),\;I_{H}^{n}=\phi_{2}\left(t_{n}\right),\;R_{H}^{n}=\phi_{3}\left(t_{n}\right),$$

$$S_{R}^{n}=\phi_{4}\left(t_{n}\right),\;I_{R}^{n}=\phi_{5}\left(t_{n}\right),\;R_{R}^{n}=\phi_{6}\left(t_{n}\right),\;t_{n}\in \left[-\tau ,0\right],$$

where


$$\tau =\max\left\{\tau_{H},\tau_{R}\right\}.$$

### 6.4 Positivity and boundedness

In this subsection, we discuss the positivity and boundedness of the stochastic NSFD scheme (Equations 33–38). Since the model represents human and rodent populations, the numerical solution must remain biologically meaningful. Thus, all discrete state variables should be non-negative, and the total human and rodent populations should remain bounded in an appropriate stochastic sense.

**Theorem 15** (Positivity). *Let the initial history functions satisfy*


$$S_{H}^{n},\;I_{H}^{n},\;R_{H}^{n},\;S_{R}^{n},\;I_{R}^{n},\;R_{R}^{n}\geq 0,\;n\leq 0.$$

*Assume that the stochastic correction is taken in the positivity-preserving exponential form*


$$X^{n}+\sigma_{i}X^{n}\Delta W_{i}^{n}\;\to \;X^{n}\exp\left(\sigma_{i}\Delta W_{i}^{n}-\frac{1}{2}\sigma_{i}^{2}h\right).$$

*Then the stochastic NSFD scheme preserves non-negativity, namely*


$$S_{H}^{n},\;I_{H}^{n},\;R_{H}^{n},\;S_{R}^{n},\;I_{R}^{n},\;R_{R}^{n}\geq 0\text{ a.s. for all }n\geq 0.$$

*Proof*. By using the exponential stochastic correction, the NSFD scheme can be written as


$$\begin{array}{l} S_{H}^{n+1}=\frac{S_{H}^{n}\exp\left(\sigma_{1}\Delta W_{1}^{n}-\frac{1}{2}\sigma_{1}^{2}h\right)+h\Gamma_{H}}{1+h\left(\mu_{H}+\alpha_{H}I_{R}^{n-m_{H}}\right)}, \end{array}$$ (39)

$$\begin{array}{l} I_{H}^{n+1}=\frac{I_{H}^{n}\exp\left(\sigma_{2}\Delta W_{2}^{n}-\frac{1}{2}\sigma_{2}^{2}h\right)+h\alpha_{H}S_{H}^{n-m_{H}}I_{R}^{n-m_{H}}}{1+h\left(\mu_{H}+\gamma_{H}\right)}, \end{array}$$ (40)

$$\begin{array}{l} R_{H}^{n+1}=\frac{R_{H}^{n}\exp\left(\sigma_{3}\Delta W_{3}^{n}-\frac{1}{2}\sigma_{3}^{2}h\right)+h\gamma_{H}I_{H}^{n}}{1+h\mu_{H}}, \end{array}$$ (41)

$$\begin{array}{l} S_{R}^{n+1}=\frac{S_{R}^{n}\exp\left(\sigma_{4}\Delta W_{4}^{n}-\frac{1}{2}\sigma_{4}^{2}h\right)+h\Gamma_{R}}{1+h\left(\mu_{R}+\alpha_{R}I_{R}^{n-m_{R}}\right)}, \end{array}$$ (42)

$$\begin{array}{l} I_{R}^{n+1}=\frac{I_{R}^{n}\exp\left(\sigma_{5}\Delta W_{5}^{n}-\frac{1}{2}\sigma_{5}^{2}h\right)+h\alpha_{R}S_{R}^{n-m_{R}}I_{R}^{n-m_{R}}}{1+h\left(\mu_{R}+\gamma_{R}\right)}, \end{array}$$ (43)

$$\begin{array}{l} R_{R}^{n+1}=\frac{R_{R}^{n}\exp\left(\sigma_{6}\Delta W_{6}^{n}-\frac{1}{2}\sigma_{6}^{2}h\right)+h\gamma_{R}I_{R}^{n}}{1+h\mu_{R}}. \end{array}$$ (44)

Since


$$\exp\left(\sigma_{i}\Delta W_{i}^{n}-\frac{1}{2}\sigma_{i}^{2}h\right)>0\text{ a.s.},$$

and since all parameters and delayed state variables are non-negative, every numerator in the above equations is non-negative. Moreover, all denominators are strictly positive because


$$1+h\left(\mu_{H}+\alpha_{H}I_{R}^{n-m_{H}}\right)>0,\;1+h\left(\mu_{H}+\gamma_{H}\right)>0,\;1+h\mu_{H}>0,$$

$$1+h\left(\mu_{R}+\alpha_{R}I_{R}^{n-m_{R}}\right)>0,\;1+h\left(\mu_{R}+\gamma_{R}\right)>0,\;1+h\mu_{R}>0.$$

Therefore,


$$S_{H}^{n+1},\;I_{H}^{n+1},\;R_{H}^{n+1},\;S_{R}^{n+1},\;I_{R}^{n+1},\;R_{R}^{n+1}\geq 0\text{ a.s.}$$

By induction, the result holds for all *n* ≥ 0.

**Theorem 16** (Boundedness in expectation). *Let*


$$N_{H}^{n}=S_{H}^{n}+I_{H}^{n}+R_{H}^{n},\;N_{R}^{n}=S_{R}^{n}+I_{R}^{n}+R_{R}^{n}.$$

*Then the stochastic NSFD scheme is bounded in expectation. In particular, there exist positive constants *M*_*H*_ and *M*_*R*_ such that*


$$\underset{n\to \infty }{lim sup}𝔼\left[N_{H}^{n}\right]\leq M_{H},\;\underset{n\to \infty }{lim sup}𝔼\left[N_{R}^{n}\right]\leq M_{R}.$$

*Proof*. For the human population, the continuous model satisfies


$$dN_{H}\left(t\right)=\left[\Gamma_{H}-\mu_{H}N_{H}\left(t\right)\right]dt+\sigma_{1}S_{H}\left(t\right)dW_{1}\left(t\right)+\sigma_{2}I_{H}\left(t\right)dW_{2}\left(t\right)$$

$$+\sigma_{3}R_{H}\left(t\right)dW_{3}\left(t\right).$$

The NSFD discretization treats the removal terms non-locally. Summing the human compartments and taking expectations, while using


$$𝔼\left[\Delta W_{i}^{n}\right]=0,$$

gives the discrete inequality


$$𝔼\left[N_{H}^{n+1}\right]\leq \frac{𝔼\left[N_{H}^{n}\right]+h\Gamma_{H}}{1+h\mu_{H}}.$$

Let


$$Y_{n}=𝔼\left[N_{H}^{n}\right].$$

Then


$$Y_{n+1}\leq \frac{Y_{n}+h\Gamma_{H}}{1+h\mu_{H}}.$$

Solving this first-order difference inequality gives


$$Y_{n}\leq {\left(\frac{1}{1+h\mu_{H}}\right)}^{n}Y_{0}+\frac{\Gamma_{H}}{\mu_{H}}\left[1-{\left(\frac{1}{1+h\mu_{H}}\right)}^{n}\right].$$

Hence,


$$\underset{n\to \infty }{lim sup}𝔼\left[N_{H}^{n}\right]\leq \frac{\Gamma_{H}}{\mu_{H}}.$$

Thus, one may take


$$M_{H}=\frac{\Gamma_{H}}{\mu_{H}}.$$

Similarly, for the rodent population, summing the rodent compartments gives


$$𝔼\left[N_{R}^{n+1}\right]\leq \frac{𝔼\left[N_{R}^{n}\right]+h\Gamma_{R}}{1+h\mu_{R}}.$$

Let


$$Z_{n}=𝔼\left[N_{R}^{n}\right].$$

Then


$$Z_{n+1}\leq \frac{Z_{n}+h\Gamma_{R}}{1+h\mu_{R}}.$$

Solving this inequality yields


$$Z_{n}\leq {\left(\frac{1}{1+h\mu_{R}}\right)}^{n}Z_{0}+\frac{\Gamma_{R}}{\mu_{R}}\left[1-{\left(\frac{1}{1+h\mu_{R}}\right)}^{n}\right].$$

$$\underset{n\to \infty }{lim sup}𝔼\left[N_{R}^{n}\right]\leq \frac{\Gamma_{R}}{\mu_{R}}.$$

Thus, one may take


$$M_{R}=\frac{\Gamma_{R}}{\mu_{R}}.$$

Hence, the stochastic NSFD scheme is bounded in expectation.

## 7 Graphical simulation

The graphical simulations illustrate the dynamical behavior of the proposed stochastic delay hantavirus model under deterministic and stochastic settings. The simulations compare the Euler–Maruyama method, stochastic Runge–Kutta method, stochastic NSFD scheme, and the corresponding deterministic model. The model describes hantavirus transmission among rodents and spillover infection from rodents to humans. Therefore, the infected rodent class *I*_*R*_(*t*) is treated as the main reservoir-driving compartment. The parameter values used in the numerical simulations are listed in Table 3.

**Table 3:** Parameter values used in the stochastic delay hantavirus model.

Parameter	Value
*b*	0.1
β_*H*_	0.0015
Γ_*H*_	0.25
γ_*H*_	$\frac{1}{200}$
μ_*H*_	$\frac{1}{65\times 365}$
β_*R*_	0.002
Γ_*R*_	0.25
γ_*R*_	0.0075
μ_*R*_	0.007
η_*H*_	0.01
η_*R*_	0.01

The initial conditions used for the simulations are


$$S_{H}\left(0\right)=1000,\;I_{H}\left(0\right)=2,\;R_{H}\left(0\right)=0,\;$$

$$S_{R}\left(0\right)=50,\;I_{R}\left(0\right)=5,\;R_{R}\left(0\right)=0.$$

The delay terms are evaluated using the prescribed history functions over [−τ, 0], where


$$\tau =\max\left\{\tau_{H},\tau_{R}\right\}.$$

### 7.1 Discussion

**Figure 3:**
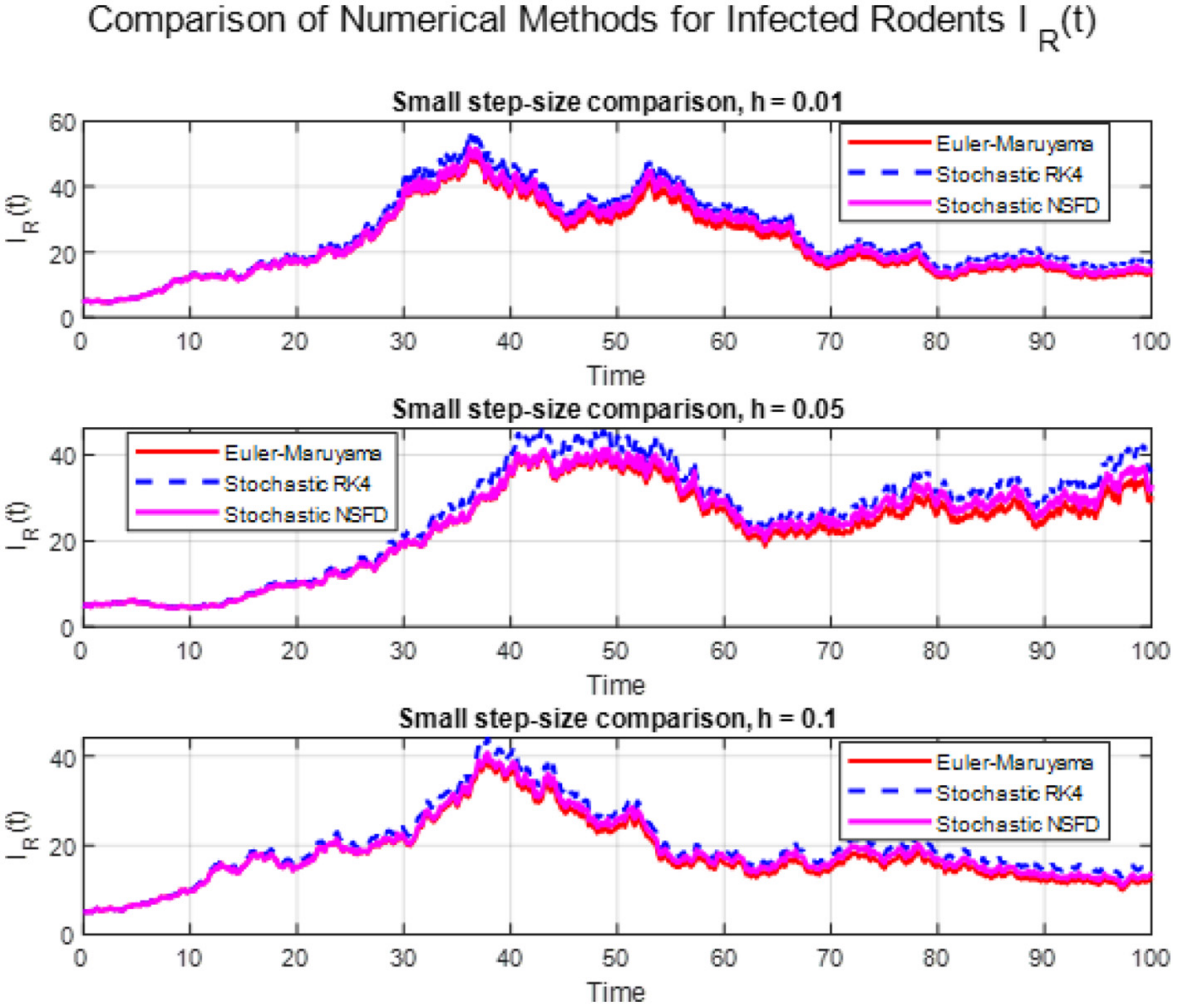
Comparison of Euler–Maruyama, stochastic Runge–Kutta, and stochastic NSFD methods for the infected rodent population *I*_*R*_(*t*) under small step sizes.

Figure 3 compares the Euler–Maruyama, stochastic RK4, and stochastic NSFD methods for the infected rodent population *I*_*R*_(*t*) under small step sizes *h* = 0.01, *h* = 0.05, and *h* = 0.1. For all three step sizes, the infected rodent population follows a similar qualitative pattern. It starts from the initial value *I*_*R*_(0) = 5, increases to a peak, and then gradually decreases. For *h* = 0.01, the peak is approximately 50 and occurs around *t* = 35–40. For *h* = 0.05, the peak is approximately 40–45, while for *h* = 0.1 the peak remains close to 40. The three numerical methods are in close agreement for these small step sizes, indicating that the schemes are numerically consistent when the time discretization is sufficiently fine.

**Figure 4:**
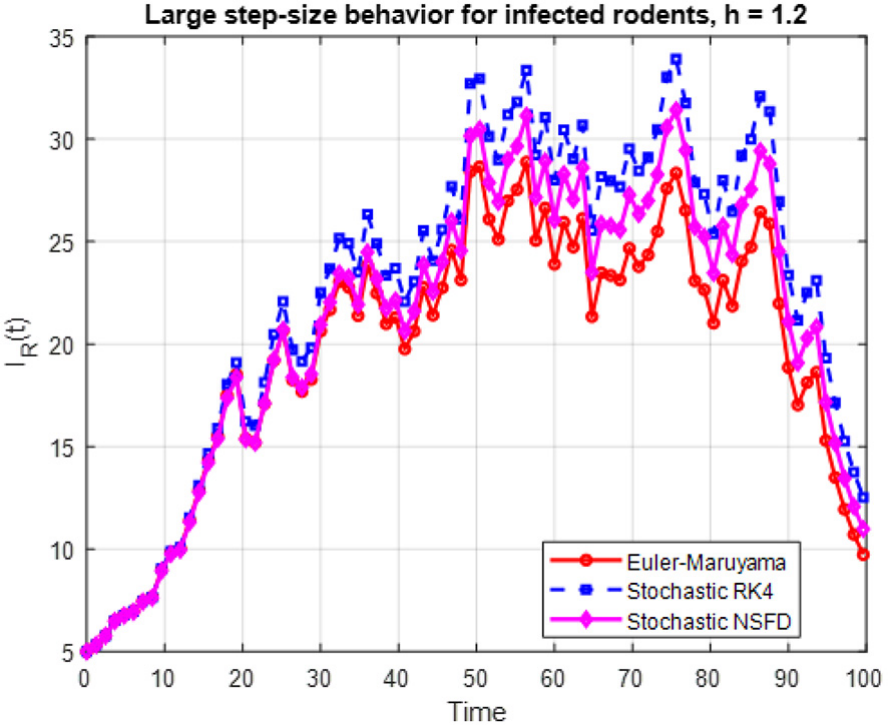
Large-step-size comparison of Euler–Maruyama, stochastic Runge–Kutta, and stochastic NSFD methods for *I*_*R*_(*t*).

However, the behavior of the numerical schemes at larger time steps, as shown in Figure 4 for *h* = 1.2, indicates that larger oscillations emerge in the infected rodent population. Under this coarse discretization, the stochastic RK4 method shows the strongest fluctuations, reaching values of approximately 30–35. The Euler–Maruyama method shows smaller fluctuations, generally around 10–15. In contrast, the stochastic NSFD trajectory remains comparatively smoother and preserves non-negativity. This confirms that the NSFD scheme provides a more biologically feasible and numerically stable approximation when larger step sizes are used.

**Figure 5:**
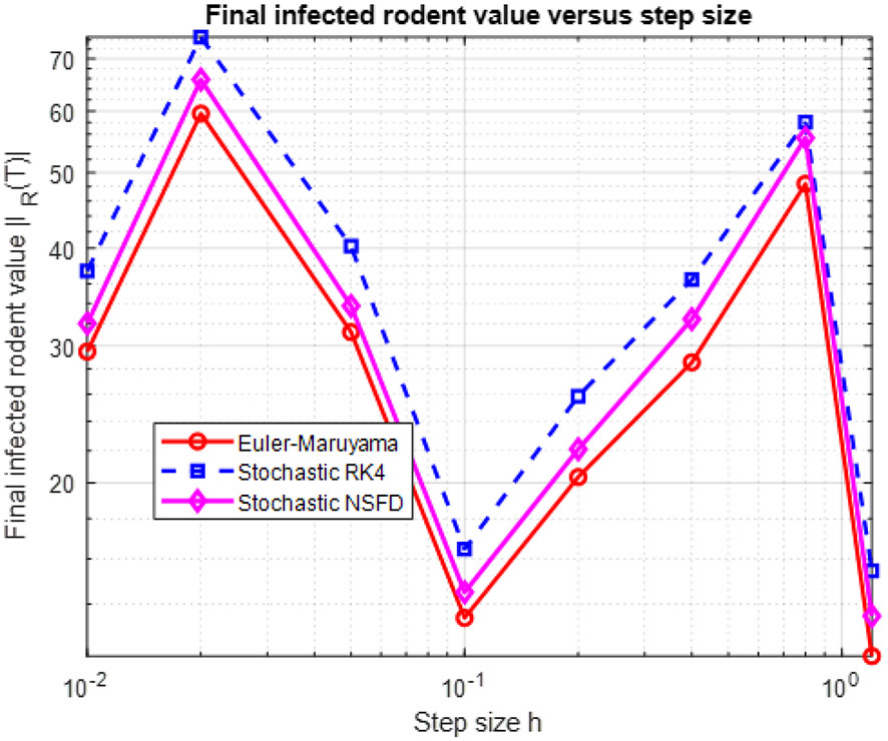
Final infected rodent value |*I*_*R*_(*T*)| obtained by Euler–Maruyama, stochastic Runge–Kutta, and stochastic NSFD methods for different step sizes.

Figure 5 displays the final infected rodent value |*I*_*R*_(*T*)| for different step sizes. Because Brownian perturbations are included in each stochastic simulation, the final infection level changes irregularly with the step size. In some cases, the stochastic RK4 method produces relatively large terminal values, reaching approximately 60–70. The Euler–Maruyama and stochastic NSFD methods also show step-size dependence, but the NSFD values remain more controlled in comparison. These results indicate that the final infection level is sensitive to both the selected numerical method and the time-step size.

**Figure 6:**
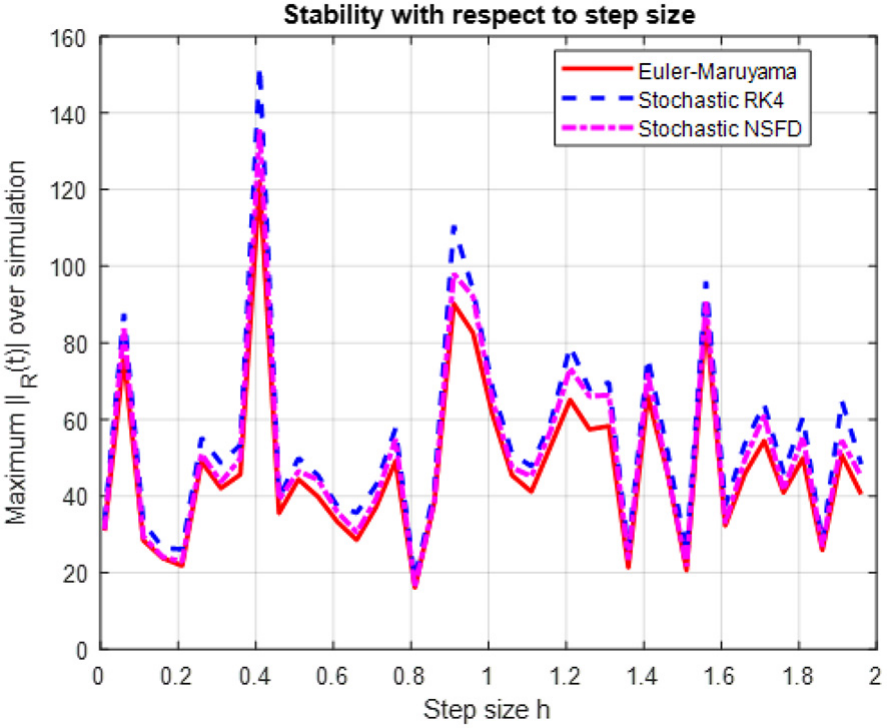
Maximum infected rodent population over the simulation interval for different step sizes, comparing Euler–Maruyama, stochastic Runge–Kutta, and stochastic NSFD methods.

Figure 6 shows the maximum value of the infected rodent population over the full simulation interval for different step sizes ranging from *h* = 0.01 to *h* = 2.0. The maximum value varies from approximately 20 to nearly 150, depending on the method and step size. The largest amplitude is observed near *h* = 0.4, particularly for the stochastic RK4 method. Although Euler–Maruyama and stochastic NSFD also show increased peak values for some step sizes, the NSFD method remains comparatively more stable. This demonstrates that classical stochastic schemes can be highly sensitive to step-size changes, whereas the NSFD scheme provides improved qualitative stability.

**Figure 7:**
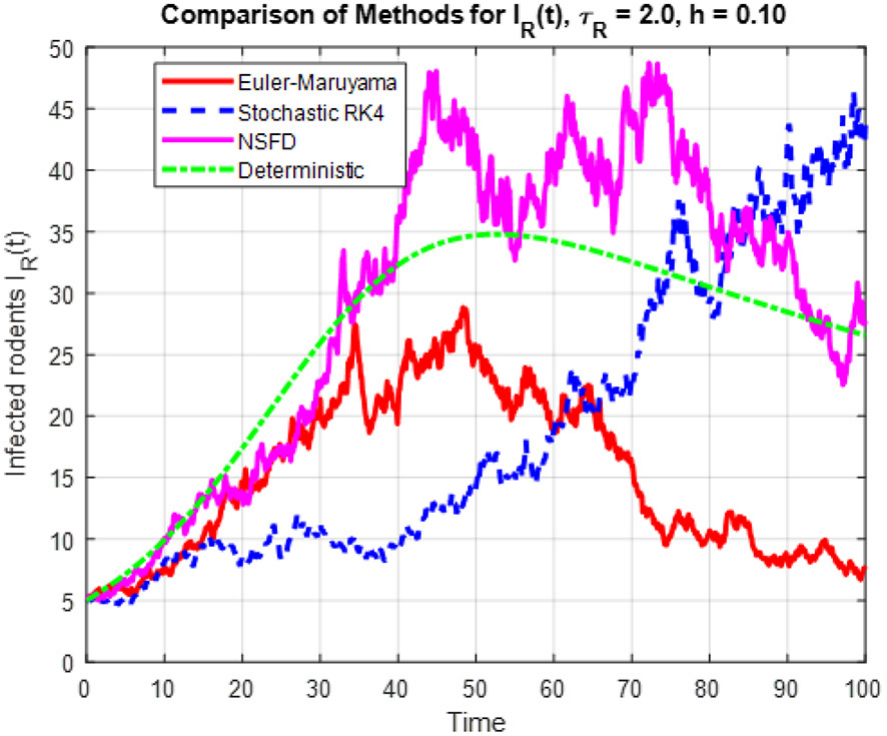
Comparison between the deterministic solution and stochastic numerical methods for the infected rodent population *I*_*R*_(*t*).

Figure 7 compares the deterministic solution with the stochastic numerical methods for *I*_*R*_(*t*). The deterministic curve is smooth and reaches a peak of approximately 35. The stochastic trajectories follow the same general trend but fluctuate around the deterministic solution because of environmental noise. In particular, stochastic perturbations can temporarily increase the infected rodent population above the deterministic prediction. This shows that random environmental effects may amplify infection levels over short time intervals, even when the long-term trend remains decreasing.

**Figure 8:**
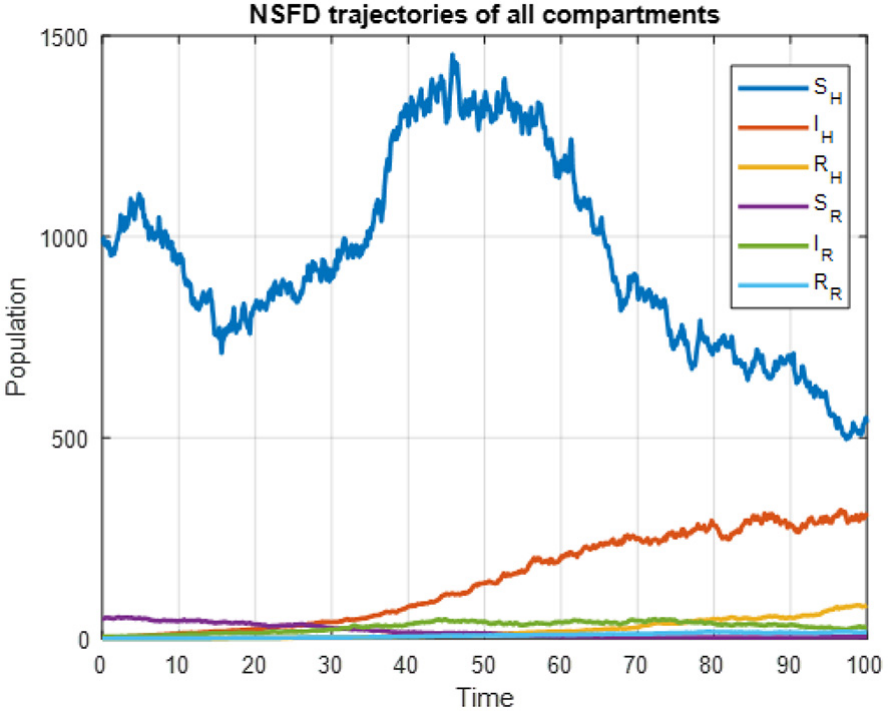
NSFD trajectories of all human and rodent compartments in the stochastic delay hantavirus model.

Figure 8 presents the stochastic NSFD trajectories of all human and rodent compartments. The susceptible human population *S*_*H*_(*t*) remains the largest compartment and fluctuates approximately between 500 and 1, 400. The infected human population *I*_*H*_(*t*) increases gradually and reaches nearly 300 by the end of the simulation. The infected rodent population *I*_*R*_(*t*) remains much smaller, mostly below 50, but it still sustains human spillover infection. This suggests that even a moderate level of infection in the rodent reservoir may maintain a considerable human infection burden through repeated exposure.

**Figure 9:**
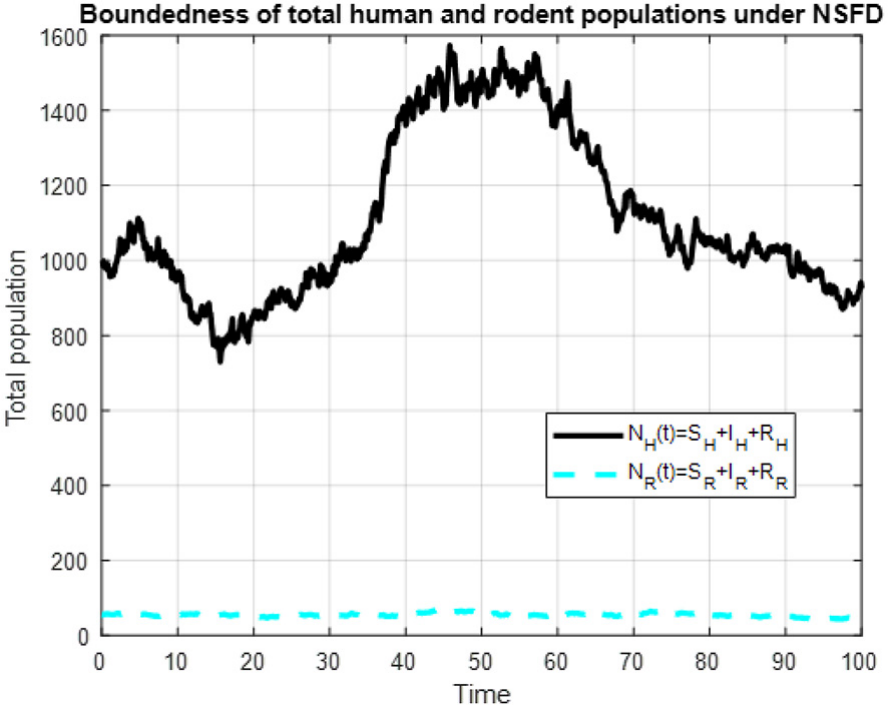
Boundedness of the total human population *N*_*H*_(*t*) and total rodent population *N*_*R*_(*t*) under the stochastic NSFD scheme.

Figure 9 illustrates the boundedness of the total human and rodent populations under the stochastic NSFD scheme. The total human population *N*_*H*_(*t*) remains approximately between 750 and 1, 550, while the total rodent population *N*_*R*_(*t*) stays around 50–80. These bounded trajectories support the theoretical boundedness result and show that the NSFD scheme preserves biologically meaningful population sizes in the presence of stochastic fluctuations.

**Figure 10:**
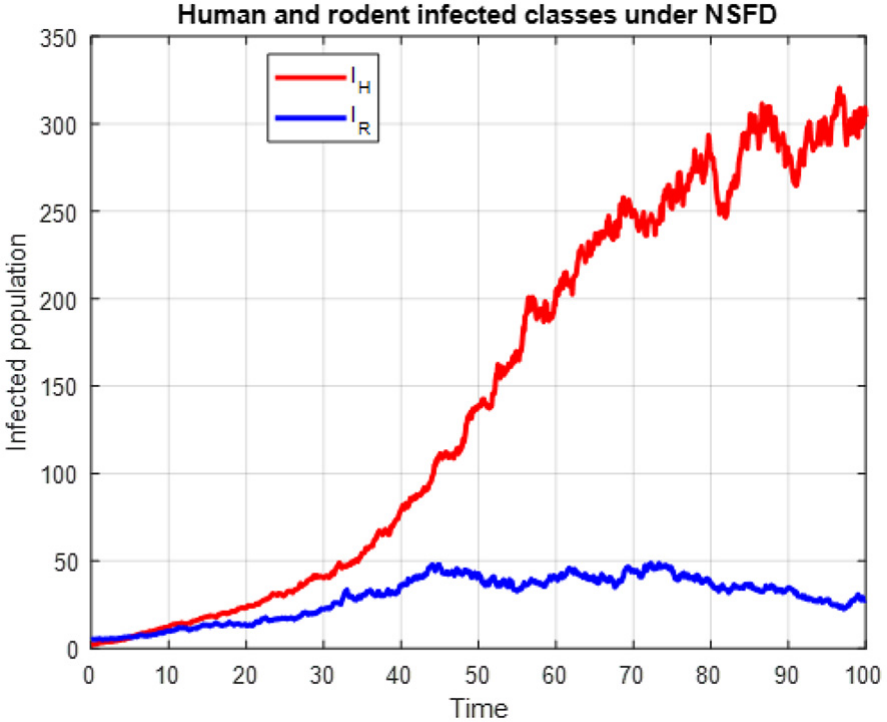
Comparison of infected humans *I*_*H*_(*t*) and infected rodents *I*_*R*_(*t*) under the stochastic NSFD scheme.

**Figure 11:**
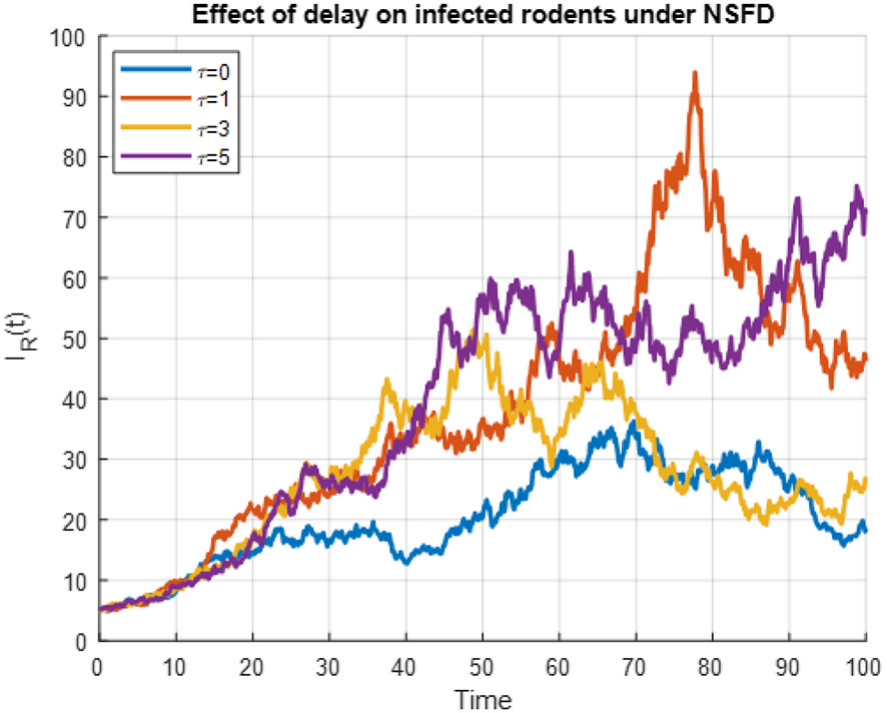
Effect of transmission delay τ on the infected rodent population *I*_*R*_(*t*) under the stochastic NSFD method.

Figure 10 compares the infected human population *I*_*H*_(*t*) and the infected rodent population *I*_*R*_(*t*) under the stochastic NSFD method. The infected rodent population increases from 5 to approximately 45–50 and then decreases slightly. By contrast, the infected human population increases steadily and reaches about 300 by the final time. This behavior indicates that human infection can continue to increase even when the infected rodent population remains moderate, because humans repeatedly receive spillover exposure from infected rodents. Figure 11 shows the effect of different transmission delay values on the infected rodent population *I*_*R*_(*t*). When τ = 0, the infected rodent population reaches a moderate peak of about 35. For τ = 1, the infection rises sharply and reaches nearly 90, giving the largest outbreak among the tested delay values. For τ = 3, the peak is approximately 50, whereas for τ = 5, the curve increases more slowly and approaches about 70 near the end of the simulation. These results show that the delay parameter affects both the timing and magnitude of the infection peak.

**Figure 12:**
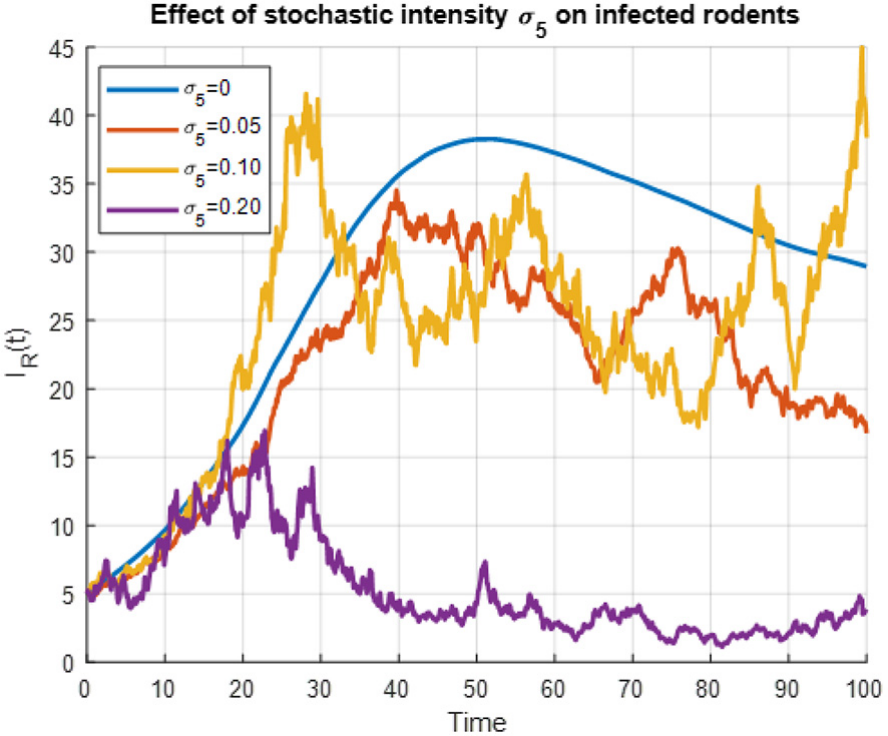
Effect of infected-rodent noise intensity σ_5_ on the infected rodent population *I*_*R*_(*t*) under the stochastic NSFD method.

Figure 12 examines the effect of the infected-rodent noise intensity σ_5_ on *I*_*R*_(*t*). When σ_5_ = 0, the trajectory is smooth and peaks near 35–40. For σ_5_ = 0.05 and σ_5_ = 0.10, stronger stochastic variation is observed, and the infected rodent population may reach higher temporary peaks. When σ_5_ = 0.20, the infected rodent population declines rapidly and remains mostly below 10 after the early phase. This supports the theoretical threshold result that sufficiently strong noise in the infected rodent class can reduce persistence and promote extinction.

**Figure 13:**
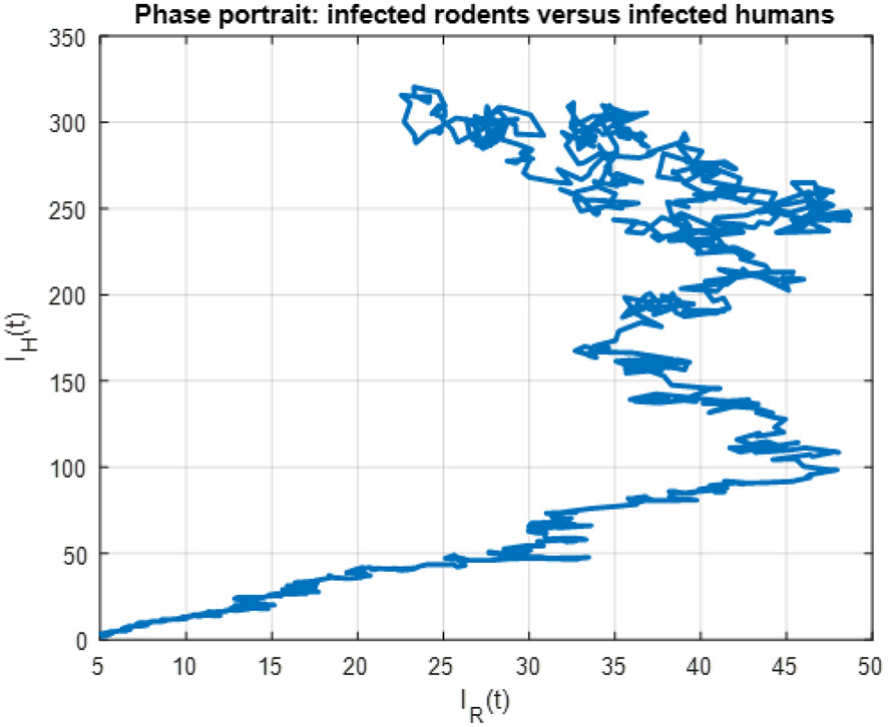
Phase portrait between infected rodents *I*_*R*_(*t*) and infected humans *I*_*H*_(*t*).

Figure 13 presents the phase relationship between the infected rodent population *I*_*R*_(*t*) and the infected human population *I*_*H*_(*t*). The trajectory starts near (*I*_*R*_, *I*_*H*_) = (5, 2) and moves upward as both infected populations increase. Later, *I*_*H*_(*t*) continues to rise while *I*_*R*_(*t*) fluctuates between approximately 25 and 50. This indicates a delayed spillover effect, where human infection may continue to increase even after the infected rodent population begins to fluctuate or decline.

**Figure 14:**
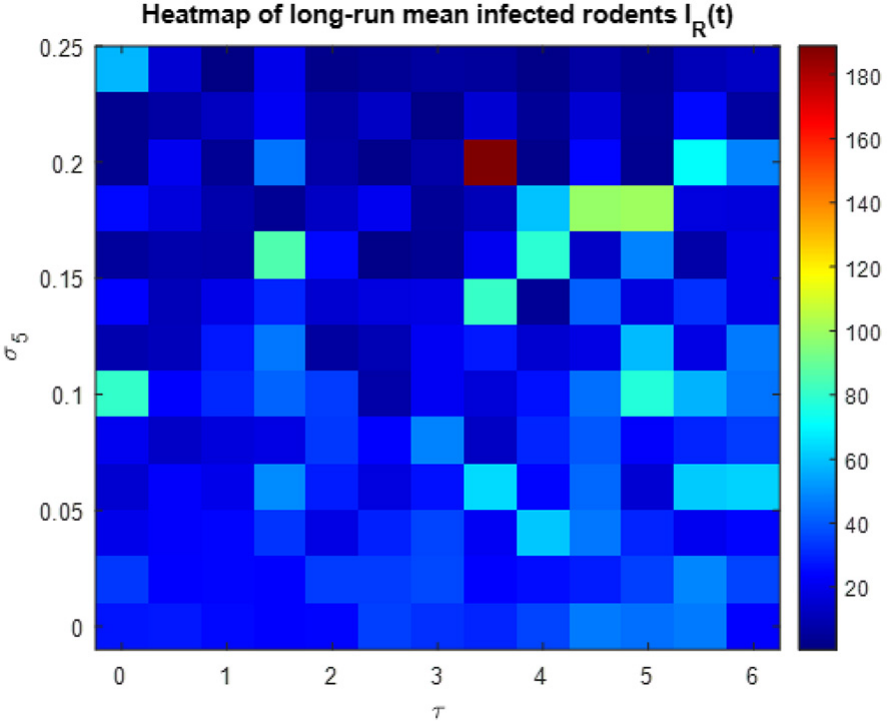
Heatmap of the long-run mean infected rodent population *I*_*R*_(*t*) for different values of delay τ and infected-rodent noise intensity σ_5_.

Figure 14 shows the long-run mean value of *I*_*R*_(*t*) for different combinations of the delay τ and the noise intensity σ_5_. Most parameter regions show low or moderate infection levels, generally below 60. However, some combinations produce higher values, exceeding 100 and approaching about 180. This indicates that delay and stochasticity may interact nonlinearly and create localized regions of stronger disease persistence.

**Figure 15:**
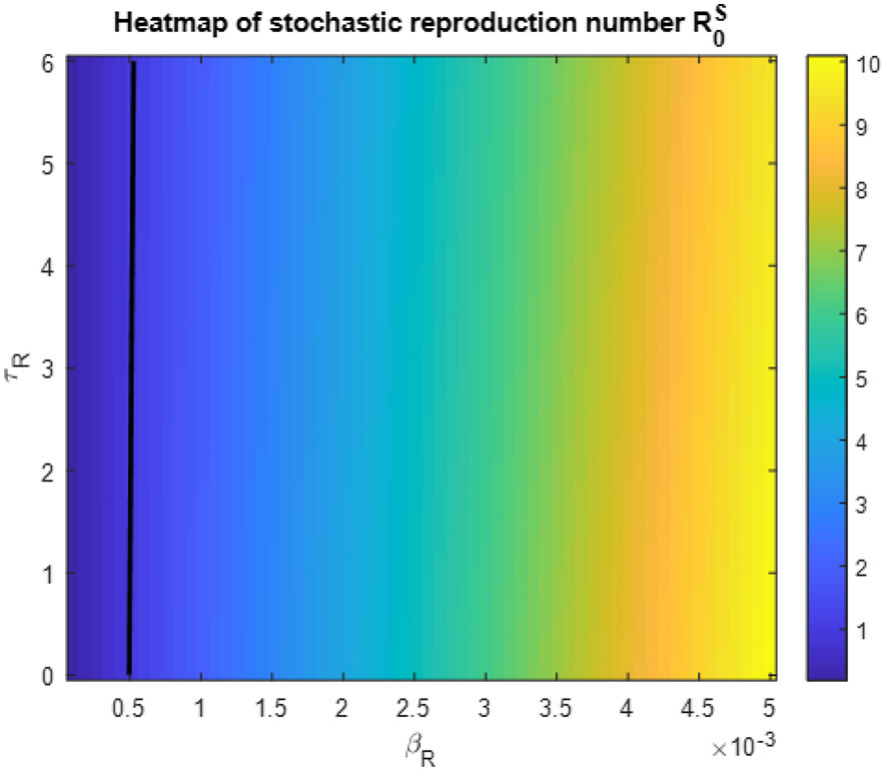
Heatmap of the stochastic reproduction number $R_{0}^{S}$ with respect to the rodent transmission rate β_*R*_ and rodent transmission delay τ_*R*_. The black line represents the threshold contour $R_{0}^{S}=1$, which separates the extinction and persistence regions.

Figure 15 presents the stochastic reproduction number ${ℛ}_{0}^{S}$ as a function of the rodent transmission rate β_*R*_ and the rodent transmission delay τ_*R*_. The value of ${ℛ}_{0}^{S}$ increases strongly as β_*R*_ increases. For small values of β_*R*_, around 0.5 × 10^−3^, ${ℛ}_{0}^{S}$ is close to or below unity. When β_*R*_ approaches 5 × 10^−3^, ${ℛ}_{0}^{S}$ increases to nearly 10. This confirms that rodent-to-rodent transmission is the main driver of disease persistence in the proposed model.

**Figure 16:**
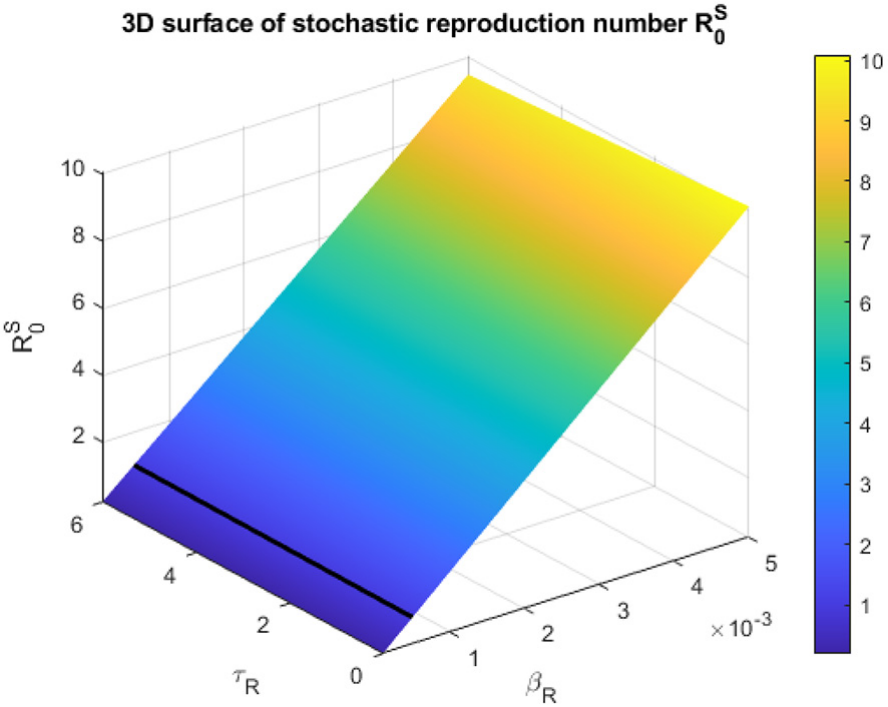
Three-dimensional surface of the stochastic reproduction number $R_{0}^{S}$ as a function of the rodent transmission rate β_*R*_ and rodent transmission delay τ_*R*_. The black line represents the threshold contour $R_{0}^{S}=1$, which separates the extinction and persistence regions.

Figure 16 gives the three-dimensional surface corresponding to the reproduction-number heatmap. The surface rises steeply in the direction of increasing β_*R*_, while increasing τ_*R*_ slightly reduces ${ℛ}_{0}^{S}$ through the exponential delay factor. The surface separates the low-risk region, where ${ℛ}_{0}^{S}<1$, from the persistence region, where ${ℛ}_{0}^{S}>1$. This confirms that reducing the rodent transmission rate β_*R*_ is more effective than relying only on delay effects.

**Figure 17:**
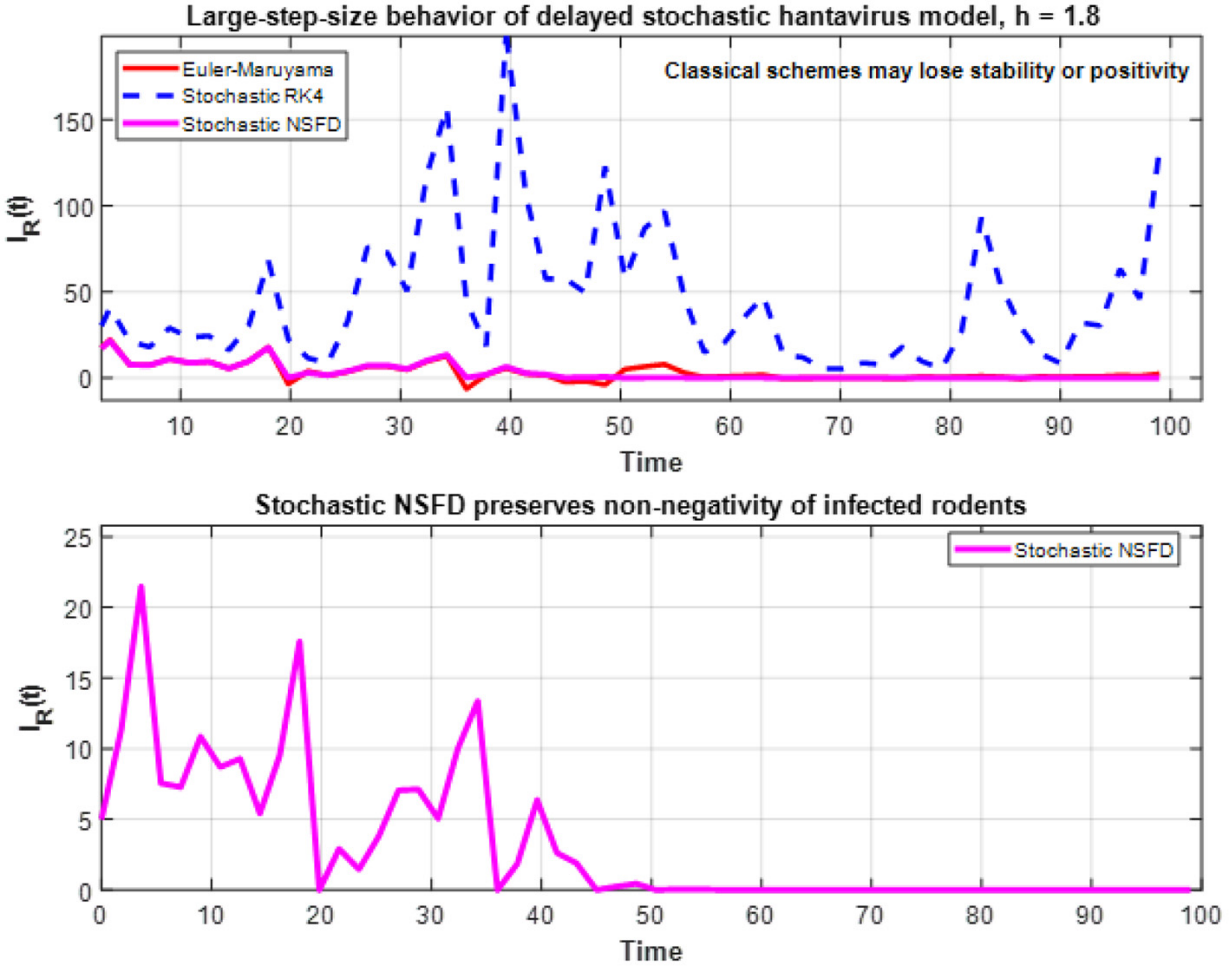
Large-step-size comparison of Euler–Maruyama, stochastic Runge–Kutta, and stochastic NSFD methods under strong noise in the infected rodent class. The first panel compares all three numerical methods, while the second panel isolates the stochastic NSFD trajectory to show its non-negative and stable behavior more clearly.

Figure 17 shows the behavior of the numerical methods under a large step size *h* = 1.8 and strong noise in the infected rodent class. Euler–Maruyama and stochastic RK4 display unstable oscillations, with stochastic RK4 reaching values above 150. In contrast, the stochastic NSFD solution remains non-negative and eventually declines toward zero after approximately *t* = 50. The lower panel isolates the NSFD curve, showing more clearly that the method preserves biological feasibility and maintains stable behavior under strong stochastic perturbations.

Overall, the numerical results confirm that the infected rodent population is the main reservoir-driving component of the model. The simulations show that rodent-to-rodent transmission strongly increases the persistence potential of hantavirus, whereas delay effects and stochastic noise may alter the timing, magnitude, and long-term behavior of infection. The comparison of numerical methods further shows that the stochastic NSFD scheme is more suitable for long-term simulation because it preserves non-negativity and provides stable biologically meaningful trajectories, especially under large step sizes and strong stochastic perturbations.

## 8 Conclusion

This study developed and analyzed a stochastic delay model for hantavirus transmission between human and rodent populations. The model was constructed from an SIR–SIR framework, where rodents act as the main reservoir and humans become infected through spillover transmission. To make the system more realistic, stochastic perturbations, discrete delays, and exponential delay-survival terms were incorporated into the model formulation. The qualitative analysis showed that the proposed system is biologically meaningful. Positivity and boundedness results confirmed that the human and rodent populations remain non-negative and finite under suitable initial conditions. The disease-free equilibrium was obtained, and the endemic equilibrium was shown to exist when the delayed reproduction threshold exceeds unity. The basic reproduction number and stochastic reproduction number were derived, showing that disease persistence is mainly controlled by the rodent-to-rodent transmission rate β_*R*_, rodent recruitment Γ_*R*_, rodent mortality μ_*R*_, recovery rate γ_*R*_, delay factor $e^{-\eta_{R}\tau_{R}}$, and infected-rodent noise intensity σ_5_. The extinction and persistence analysis demonstrated that hantavirus dies out when the stochastic reproduction number satisfies ${ℛ}_{0}^{S}<1$, whereas persistence is expected when ${ℛ}_{0}^{S}>1$. The stochastic threshold also showed that stronger noise in the infected rodent class can reduce the effective persistence potential, while higher rodent-to-rodent transmission increases the likelihood of sustained infection. Real-data analysis using reported 2026 hantavirus updates supported the relevance of the model. The February–May 2026 data showed continued hantavirus activity, especially in Argentina, and also highlighted travel-associated outbreak risk. Although the available data were reported as public-health update points rather than exact monthly incidence, they provided useful motivation for studying reservoir-driven transmission and stochastic effects. Three numerical methods were considered: Euler–Maruyama, stochastic Runge–Kutta, and stochastic nonstandard finite difference schemes. The numerical simulations showed that all methods behave consistently for small step sizes. However, for large step sizes and strong noise, Euler–Maruyama and stochastic RK4 may produce unstable or biologically unrealistic oscillations. In contrast, the stochastic NSFD scheme preserved non-negativity and produced more stable trajectories, making it more suitable for long-term simulation of stochastic epidemic models. The graphical simulations also supported that the major factor in the persistence of hantavirus is rodent infection. The heatmap and three dimensional surface of ${ℛ}_{0}^{S}$ revealed that indeed, raising β_*R*_ significantly raises the level of disease persistence; other parameters, such as delay effects and stochastic noise, can lower the reproduction threshold. The results indicate that controlling hantavirus transmission effectively should target primarily the reduction in rodent to rodent transmission and the exposure of humans to infected rodents and contaminated areas. The study suggests that stochastic delay models are a useful tool to gain insight into the dynamics of hantavirus in a variable environment with delayed transmission. A limitation of the present numerical analysis is that the stochastic simulations are mainly presented as representative sample paths. In future work, Monte Carlo simulations will be performed to generate ensembles of stochastic trajectories and to construct prediction intervals for infected rodent and human populations. Such an approach would provide a more complete probabilistic assessment of hantavirus spillover risk and would allow the uncertainty in long-term predictions to be quantified more rigorously.

## Data Availability

The original contributions presented in the study are included in the article/supplementary material, further inquiries can be directed to the corresponding author.
